# A novel integrated approach to predicting cancer immunotherapy efficacy

**DOI:** 10.1038/s41388-023-02670-1

**Published:** 2023-04-26

**Authors:** Ruihan Luo, Jacqueline Chyr, Jianguo Wen, Yanfei Wang, Weiling Zhao, Xiaobo Zhou

**Affiliations:** 1 West China Biomedical Big Data Center, West China Hospital, Sichuan University, Chengdu, China; 2 Med-X Center for Informatics, Sichuan University, Chengdu, China; 3 Center for Computational Systems Medicine, School of Biomedical Informatics, The University of Texas Health Science Center at Houston, Houston, TX USA; 4 McGovern Medical School, The University of Texas Health Science Center at Houston, Houston, TX USA; 5 School of Dentistry, The University of Texas Health Science Center at Houston, Houston, TX USA

**Keywords:** Immunosurveillance, Cancer genomics

## Abstract

Immunotherapies have revolutionized cancer treatment modalities; however, predicting clinical response accurately and reliably remains challenging. Neoantigen load is considered as a fundamental genetic determinant of therapeutic response. However, only a few predicted neoantigens are highly immunogenic, with little focus on intratumor heterogeneity (ITH) in the neoantigen landscape and its link with different features in the tumor microenvironment. To address this issue, we comprehensively characterized neoantigens arising from nonsynonymous mutations and gene fusions in lung cancer and melanoma. We developed a composite NEO2IS to characterize interplays between cancer and CD8+ T-cell populations. NEO2IS improved prediction accuracy of patient responses to immune-checkpoint blockades (ICBs). We found that TCR repertoire diversity was consistent with the neoantigen heterogeneity under evolutionary selections. Our defined neoantigen ITH score (NEOITHS) reflected infiltration degree of CD8+ T lymphocytes with different differentiation states and manifested the impact of negative selection pressure on CD8+ T-cell lineage heterogeneity or tumor ecosystem plasticity. We classified tumors into distinct immune subtypes and examined how neoantigen-T cells interactions affected disease progression and treatment response. Overall, our integrated framework helps profile neoantigen patterns that elicit T-cell immunoreactivity, enhance the understanding of evolving tumor-immune interplays and improve prediction of ICBs efficacy.

## Introduction

Immunotherapy has been an essential component of cancer treatment in recent decades [1]. Many prominent breakthroughs have been made in the field of cancer immunotherapy, especially the discovery of immune-checkpoint blockade (ICB) inhibitors targeting cytotoxic T-lymphocyte-associated protein (CTLA-4) and programmed cell death protein 1/programmed cell death protein ligand 1 (PD1/PDL1) [1]. Over the past decade, anti-PD1/PDL1 blockades have demonstrated remarkable clinical efficacy in non-small-cell lung cancer (NSCLC) and melanoma. Currently, tumor mutation burden (TMB) and PDL1 expression have been widely applied as important biomarkers of ICB treatment response and are often used as validated indicators to assist clinical decisions [2, 3]. Unfortunately, due to complex resistance mechanisms and a lack of consensus on cut-off values, they are insufficient to accurately predict clinical benefit of immunotherapy [4]. In addition to ICB therapies, adoptive T cell therapy and tumor vaccines are also common forms of immunotherapies. The basic mechanism is that T cells can destroy tumor cells via recognition of tumor neoantigens presented by the major histocompatibility complex (MHC) molecules [5]. Neopeptides generated by tumor-specific nonsynonymous mutations (NSMs) are ideal immunotherapy targets as they can be recognized as foreign proteins and elicit a neoantigen-specific cytotoxic T-cell response [6]. Therefore, beyond TMB and PDL1 expression, tumor neoantigen load has also been considered a potential determinant of the clinical response to ICBs.

Recently, several studies have focused predominantly on NSMs-containing neoantigens identified by whole-exome sequencing (WES) data from paired tumor and normal samples [7–9]. However, these studies ignored neoantigens originating from other genetic variations across the tumor genome, such as fusion genes. Theoretically, gene fusions can serve as ideal sources of neoantigens because they can form new open reading frames (ORFs) and produce plentiful neopeptides [10]. Therefore, in this study, we comprehensively investigated the contribution of three different types of somatic mutations, namely single-nucleotide variants (SNVs), insertion and deletions (indels) and fusion genes, to T cell recognition towards neopeptides. By taking into account the immunogenicity of above three classes of neoantigens, we linked three neoantigen load scores (NLS) to immune infiltration and CD8^+^ T cell exhaustion in tumors, and calculated a composite neoantigen load score (NEO2IS) to represent immunogenic potentials of predicted neoantigens. By analyzing 5 external ICB cohort data, we demonstrated that tumors with higher NEO2IS exhibited favorable clinical efficacy of immunotherapy and this score also improved the accuracy of predictions of treatment response.

Tumor development and metastatic progression is a Darwinian evolutionary process, involving the interplay between cancer subclones and the local immune microenvironment [11]. Multiregional tumor sampling helps characterize genetic heterogeneity within individual tumors, i.e., intratumor heterogeneity or ITH [12]. Previous studies have highlighted that clonal and subclonal neoantigens do not drive equally effective antitumor immunity [13]. Recent advances in single-cell transcriptomes have been made new insights regarding dysfunctional states, spatial arrangement, and the modulation by ICB of antigen-specific CD8^+^ T cells [14]. More emergent data now consider exhausted CD8^+^ T cells (CD8^+^ Tex) as a developmental continuum, where the lineage is comprised of ICB permissive and refractory subsets termed stem-like CD8^+^ Tex progenitors and terminally differentiated cells, with progressive loss of effector functions and ultimately culminated in apoptosis [14, 15]. By evaluating each tumor’s neoantigen ITH score (NEOITHS), we found a consistency between neoantigen diversity and the heterogeneity within the CD8^+^ tumor-infiltrating lymphocytes (TILs) lineage [15], as well as associations of TCR diversity and NEOITHS with clinical outcomes of cancer patients. We further classified included tumors into four different immune subtypes and explored how selection pressures from different tumor microenvironment (TMEs) affected immune surveillance and degree of ITH delineating different T cell subpopulations. We also analyzed how neoantigen-T cells interactions as measured by the above two metrics affected disease progression and response to treatment. We believe that the NEO2IS, NEOITHS and immune subtypes from our study hold promise as potentially valuable tools for predicting clinical response to cancer immunotherapy.

## Results

### Identification of neoantigen candidates in lung cancer and melanoma

**Fig. 1:**
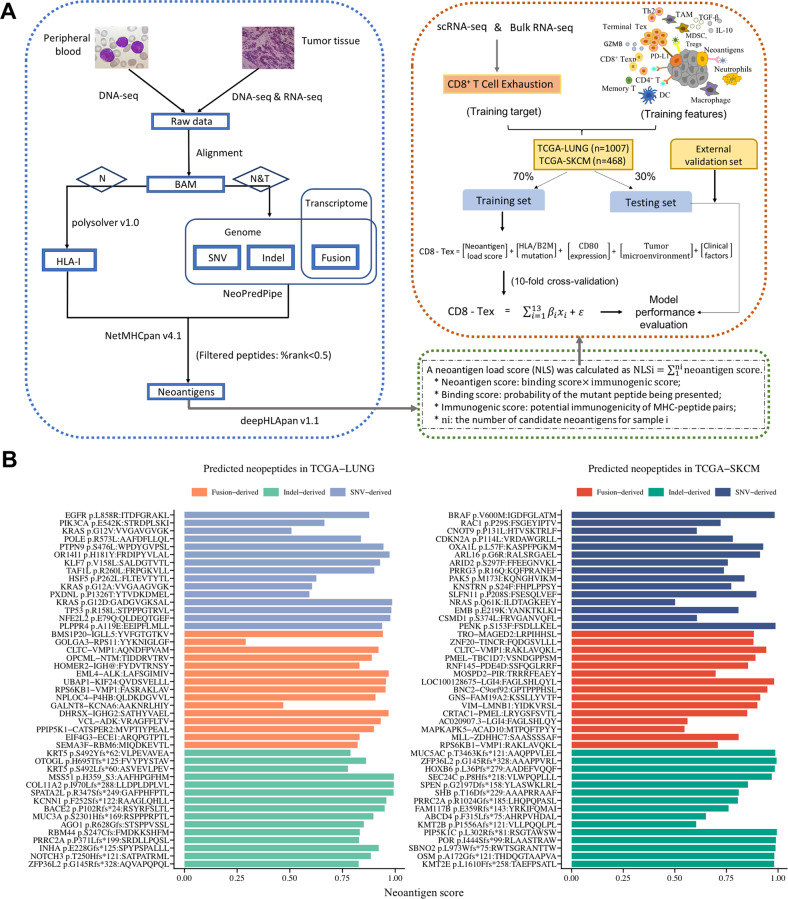
The workflow of neoantigen prediction and neoantigen load score calculation. **A** Somatic mutations and structural variants were detected using WES and RNA-seq data. Translated proteins were chopped into 8–11 kmers peptides encompassing the mutated residues (for SNVs and indels) and fusion breakpoint (for fusions) until a stop codon. Mutated peptides with binding affinity <0.5 % Rank determined by NetMHCpan were considered as candidate neoantigens. The neoantigen load score was calculated based on outputs of deepHLApan model. **B** Representative immunogenic neoantigens identified in TCGA-LUNG and TCGA-SKCM cohort.

Neopeptides arising from SNVs, indels and fusion genes were identified from TCGA WES and RNA-seq data using our screening approach (Fig. 1A). The top-ranked frequent neoantigens predicted to strongly bind to patient’s MHC class I molecules are shown in Fig. S1. Among them, the top-ranked immunogenic neoepitopes were potential targets for cancer immunotherapy, including MSS51^H359_S3^:AAFHPGFHM, KRAS^G12D^:GADGVGKSAL, TP53^R158L^:STPPPGTRVL and EML4-ALK:LAFSGIMIV identified in lung cancer (Fig. 1B); PIP5K1C^L302Rfs*81^: RSGTAWSW, BRAF^V600M^:IGDFGLATM, BNC2-C9orf92:GPTPPPHSL and CLTC-VMP1:RAKLAVQKL in melanoma (Fig. 1B). Furthermore, an indel mutation in TCGA-LUNG yielded far more neoantigen candidates than a point mutation or fusion gene did, while neoantigens derived from each of SNVs in TCGA-SKCM were more than those from each of fusions and indels (Fig. S1b).

### Association of neoantigen load scores with immune infiltration

**Fig. 2:**
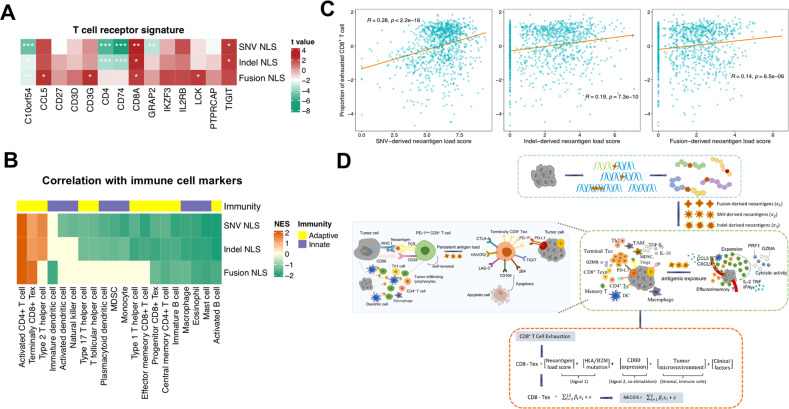
Association of neoantigen load score (NLS) with immune infiltration and construction of CD8-Tex model. **A** Heatmap depicting the correlations of mRNA expression levels of T cell receptor-related gene signatures with NLS of three types in TCGA-LUNG. Significant regression coefficient is indicated by *P < 0.05; **P < 0.01; ***P < 0.001. **B** Heatmap of GSEA normalized enrichment scores (NESs) for immune cell marker genes. Specifically, for each of three classes of NLS (fusion, SNV and indel), GSEA was performed on genes ranked by the t value from each linear regression model. Gene signatures with q-value <0.3 are retained and color-scaled. **C** The correlations of SNV-, indel- and fusion-derived NLS with CIBERSORTx-inferred exhausted CD8^+^ T cells in TCGA-LUNG samples. **D** The overview of multivariate linear regression approach modeling both tumor features and CD8^+^ T-cell exhaustion. The NLS of three types was denoted as x_1_, x_2_ and x_3_, respectively. The integrated neoantigen model (CD8-Tex) was fit to estimate the overall immunogenicity of predicted neoantigens and represent the degree of neoantigen-based CD8^+^ T-cell exhaustion in TME.

We next evaluated the relationship between the NLS and immune signatures. Multivariate linear regression analysis of gene expression levels in TCGA samples was consequently performed using each NLS together with sex, age and tumor purity. We observed positive correlations between three types of NLS and T-cell receptor-associated gene expression signatures, such as CD8A, CD3G, CCL5, TIGT, LCK and IKZF3 in lung cancers and primary melanomas after adjusting clinical factors (Figs. 2A, S2a). Interestingly, immune infiltration was found to significantly associate with high NLS, especially exhausted-like CD8^+^ T cells and activated CD4^+^ T cells (Figs. 2B, S2a).

Considering that the identified neoepitopes were all specific for CD8^+^ T lymphocytes’ recognition and significant enrichment of exhausted state of CD8^+^ T cells were observed, we then estimated the infiltration of CD8^+^ Tex based on two scRNA-seq datasets (Fig. S2b). Our results showed that three types of normalized NLS [log_2_(NLS + 1)] correlated closely with CIBERSORTx-estimated CD8^+^ Tex fractions within both TCGA-LUNG and SKCM samples (Figs. 2C, S2c). Subsequently, we constructed CD8-Tex models to examine the contributions of predicted neoantigens to immunoreactivity and CD8^+^ T-cell depletion due to persistent antigens in the TME. Based on the multivariate regression analysis, a composite NEO2IS was calculated (Fig. 2C) using NLS to delineate different differentiation status of CD8^+^ TILs at different TMEs (i.e., immunologically active or suppressive). Linear models were retained as they yielded better accuracy than SVR and GBM models when we tested them on external datasets (SMC and Abbott, Fig. S2f).

### Prediction of immunotherapy efficacy in tumors using NEO2IS

**Fig. 3:**
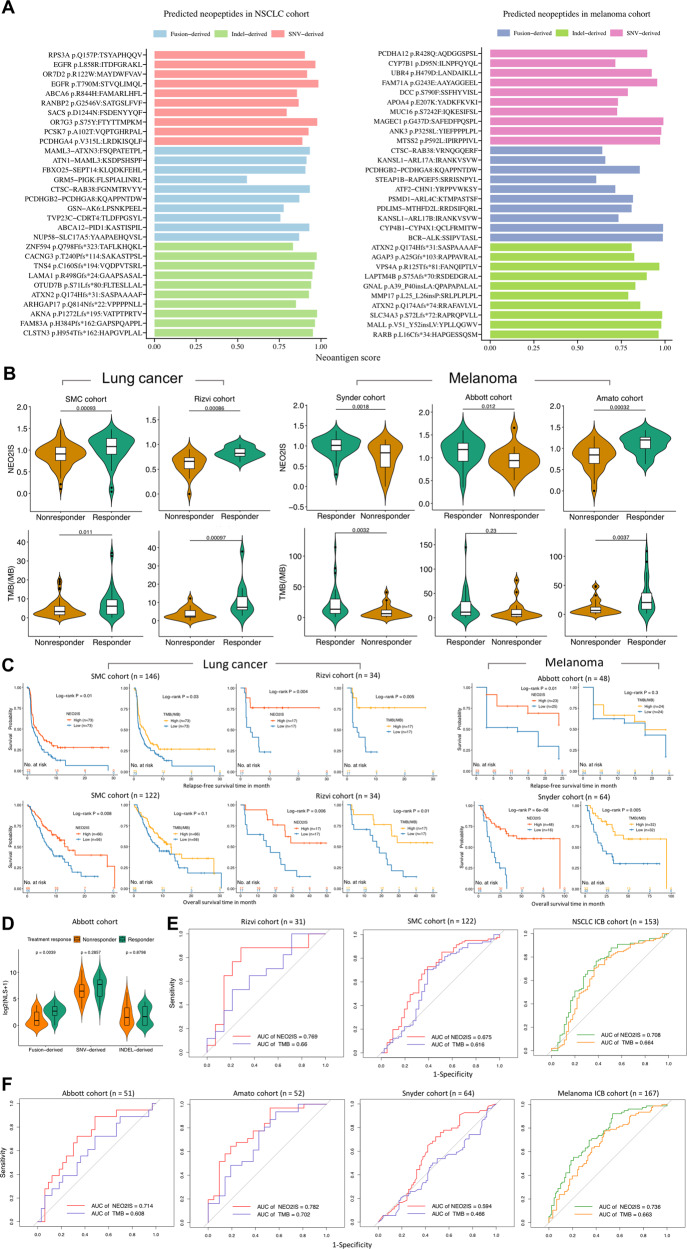
Evaluation of immunotherapeutic efficacy in tumors using the neoantigen model. **A** Overview of representative neoantigens detected in all NSCLC and melanoma samples from ICB cohorts. **B** The NEO2IS is significantly higher in responders compared with nonresponders (*p* value <0.05) and shows stronger association with treatment response than TMB in both lung cancers and melanomas. **C** Kaplan–Meier curves of RFS and OS in patients with high and low NEO2IS or TMB in lung cancer (left panel) and melanoma (right panel) ICB cohorts (quantiles or medians of cohort NEO2IS and TMB used as cut-off points). **D** Comparison of NLS between responding patients and nonresponding patients in Abbott melanoma cohort. **E** ROC curves for NEO2IS and TMB in 2 lung cancer cohorts and the combined NSCLC samples. **F** ROC curves for NEO2IS and TMB in 3 melanoma cohorts and their combined samples.

WES or bulk RNA-seq data from 2 NSCLC and 3 melanoma immunotherapy cohorts were analyzed using our neoantigen prediction pipeline. Neoantigens were highly sparse and infrequently shared between those patients. The top-ranked frequent neoantigens inferred from ICB cohorts were shown in Fig. S3. Figure 3A shows that STVQLIMQL, ITDFGRAKL and FGNMTRVYY derived from EGFR^T790M^, EGFR^L858R^, and CTSC-RAB38 respectively in NSCLC, MAGEC1^G437D^-derived SAFEDFPQSPL and BCR-ALK-derived SSIPVTASL in melanoma were the top-ranked immunogenic neoantigens. Furthermore, each SNV or an indel in both two combined immunotherapy datasets generated far more neoantigens than each fusion gene did (*p* value <0.05, Fig. S3b).

We next evaluated NEO2IS of these samples and found it significantly associated with patients’ treatment response (*p* value <0.05, Fig. 3B) and favorable survivals (log-rank *p* value <0.05, Fig. 3C). It should be noted that both higher NEO2IS and higher TMB showed significant correlation with improved clinical response in NSCLC (SMC, Rizvi) and melanoma (Amato, Snyder) cohorts (Fig. 3B, C). However, TMB failed to predict response to ICBs (*p* value = 0.23, Fig. 3B) and prognosis of melanoma patients in Abbott cohort (log-rank *p* value = 0.3, Fig. 3C) as well as overall survivals of SMC NSCLC patients (log-rank *p* value = 0.1, Fig. 3C). Fusion-derived NLSs were significantly higher in responders than non-responders, while no significant association between clinical response and SNV&indel NLS were observed in Abbott cohort (Fig. 3D). Moreover, we reasoned that our score scheme is employed to evaluate the immunogenic potential of the SNV and indel as well as the fusion-based candidate neoantigens. Therefore, NEO2IS showed a superior predictive power of clinical efficacy compared with TMB as seen from the ROC curves (Fig. 3E, F). When dichotomizing NSCLC and melanoma ICB data uniformly with cut-points of TMB > top quintile of cohort TMB and NEO2IS > median of cohort NEO2IS, respectively, we found NEO2IS could still predict prognosis of two ICB cohorts while TMB failed to stratify clinical efficacy of melanoma patients (Fig. S3c).

### Molecular features associated with neoantigen score signature in tumors

**Fig. 4:**
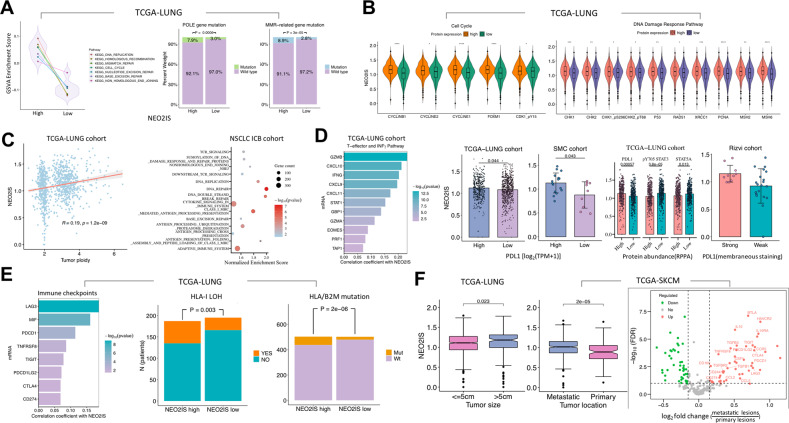
Molecular mechanisms associated with the neoantigen signature. **A** Left panel, GSVA showing DDR pathways significantly enriched in high NEO2IS group (adjusted *p* value <0.05); right panel, the estimated proportion of POLE and MMR-related gene mutations in two subgroups according to median NEO2IS. **B** Proteomic analysis showing correlation between NEO2IS and the abundance of relevant proteins involving cell cycle and DDR pathways. **C** Left panel, tumor ploidy significantly related to the neoantigen signature; right panel, GSEA enrichment results (q-value <0.3) for immunomodulatory REACTOME pathways (top 10) that correlated with NEO2IS in external NSCLC samples. **D** Comparison of expression levels of genes associated with T-effector and INFγ pathway, PDL1, pY705 STAT3 and STAT5 between high and low NEO2IS groups. **E** Potential immune evasion mechanisms associated with higher NEO2IS, including higher levels of co-inhibitory receptors, loss of heterozygosity of HLA gene locus (LOHHLA) and somatic mutations in HLA or B2M gene. **F** NEO2IS shows significant association with clinical features (left panel); differentially expressed genes between metastatic and primary tumors (right panel).

To better understand the molecular mechanisms behind above correlations, we inspected the underlying biological structures involved in immunogenicity in the TME and investigate interpretation for current intriguing observations. GSVA was performed on TCGA tumor datasets to assess the relationship between NEO2IS and molecular features. DNA replication, homologous recombination, mismatch repair (MMR), cell cycle, nucleotide excision repair, base excision repair (BER) and nonhomologous end-joining were significantly upregulated in tumors with high NEO2IS (Figs. 4A, S4a). This suggested that a high neoantigen load score was associated with the activation of the cell cycle, DNA replication and DNA damage repair (DDR) pathways.

It’s reported that improved efficacy of ICBs was independently associated with alterations in DDR pathways [16]. To determine whether NEO2IS could explain the differential pathways analyzed above, we subsequently focused on investigating the relevance of DDR pathway-related genes with the neoantigen signatures reflected by this score. We found that the mutation frequencies in POLE and MMR-related genes (including MSH2, MSH6, MLH1 and PMS2) were significantly increased in the NEO2IS-high groups (Figs. 4A, S4a). Raised abundance of proteins (TCGA RPPA data) involved in cell cycle and DDR pathways was found to significantly correlate with higher NEO2IS (Fig. 4B). Higher ploidy was also observed in tumors with higher NEO2IS (Figs. 4C, S4b). This reflected higher genomic instability and more DNA replication stress in such tumors, resulting in an increased number of neoepitopes and making them potential responders to anti-PD1 therapies [16]. Furthermore, we examined the immunogenic potential reflected in the neoantigen signatures using transcriptome data from validation cohorts. Prominent enrichment of DDR and immunomodulatory pathways was observed in both NEO2IS-high NSCLCs and melanomas (Figs. 4C, S4c). These included DNA repair, Class I MHC-mediated antigen processing and presentation, DNA double strand break repair, adaptive immune system, cytokine signaling in immune system, cytokine signaling in immune system, antigen processing: ubiquitination proteasome degradation, and TCR signaling pathway, suggesting the possible priming and expansion of neoantigen-reactive T cells against persistent antigens in those tumors. Intriguingly, mRNA expression levels of T-effector and INFγ-related gene signatures (GZMB, INFG, STAT1, CXCL9 and CXCL10) and PDL1 were also saliently associated with NEO2IS (Figs. 4D, S4c, d). Moreover, significantly increased protein abundance of PDL1, attenuated expression of pY705 STAT3 and STAT5A was related with high NEO2IS in NSCLCs (Fig. 4D). PDL1 intensity was also linked to the neoantigen signature in Rizvi samples (Fig. 4D), reflecting inflammation status or infiltrated-inflamed TME of immunologically ‘hot’ tumors [17], and potentially concomitant inhibition on antitumor T cell response through constitutively expressing PDL1 by cancer cells [18, 19].

In addition to PDL1, other inhibitory checkpoints (PDCD1, CTLA4, LAG3, TIGIT, CD160, BTLA, CD44 and HAVCR2) were also significantly correlated with the NEO2IS (Figs. 4E, S4e), implying that terminal differentiation of CD8^+^ Tex is driven by constantly elevated antigen load in tumors. More interestingly, somatic loss of heterozygosity (LOH) at the HLA-I gene locus and mutations in B2M or at least one HLA-I gene (Figs. 4F, S4e) were both associated with higher NEO2IS in NSCLCs and melanomas. These results suggest that the characteristics of high NEO2IS are more frequently presented in tumors with immune evasion or more immune-depleted TME. The growth of those tumors was thus unrestrained and became clinically apparent; for instance, higher NEO2IS was found in those with larger size and metastatic lesions (Fig. 4F). Also, numerous inhibitory checkpoints and immunosuppressive factors had increased expression in metastatic tumors compared with the primary lesion (Fig. 4F). Similar observations were reported by Braun et al. They found that PDCD1, HAVCR2 and LAG3 expression increased substantially late in pseudotime and CD8^+^ T cells became progressively dysfunctional with advancing disease in clear cell renal cell carcinoma (ccRCC) [20]. This further demonstrates the contribution of NEO2IS to characterizing interactions between cancer cells and CD8^+^ TILs.

### Neoantigen evolution and neoantigen-T cell interactions

**Fig. 5:**
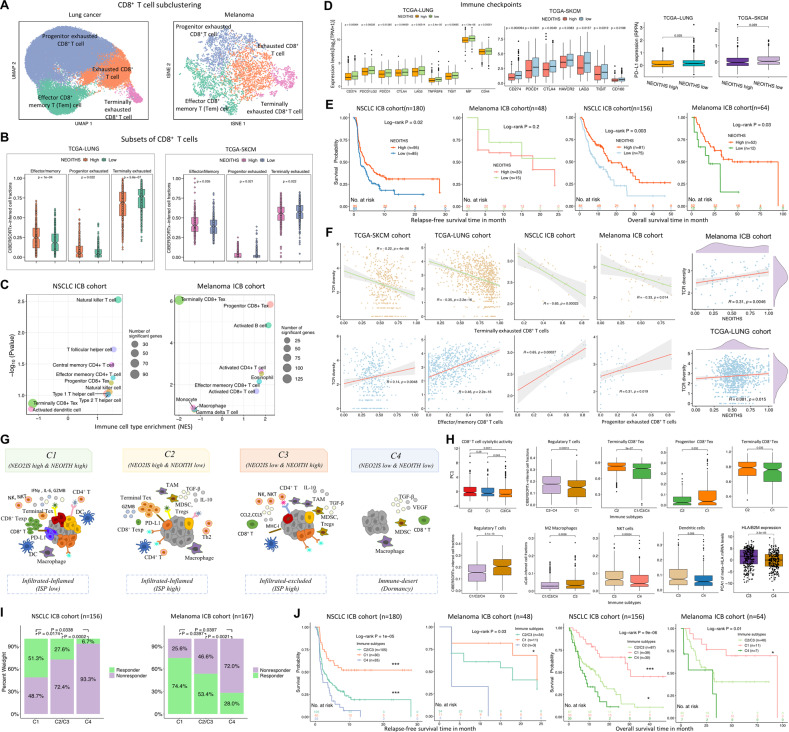
Neoantigen evolution and neoantigen-T cell interactions. **A** UMAP and tSNE of CD8^+^ T cells in lung cancers and melanomas, colored and labeled by 4 main subsets of CD8^+^ lymphocytes. **B** More fractions of effector/memory cells and progenitor CD8^+^ Tex and less terminally CD8^+^ Tex observed in TCGA tumors with higher NEOITHS (more than median cohort NEOITHS). **C** The NSCLC and melanoma ICB samples with lower NEOITHS shows increased enrichment of terminally CD8^+^ Tex while higher NEOITHS correlates with activated TILs, effector/memory CD8^+^ T cell and progenitor CD8^+^ Tex. **D** Expression levels of inhibitory checkpoints negatively correlates with NEOITHS in tumors. **E** Relapse-free survival and overall survival for NSCLC and melanoma ICB cohorts based on high NEOITHS versus low NEOITHS (mean values of cohort NEOITHS used as cut-off points). **F** Correlations between TCR repertoire diversity and proportions of CIBERSORTx-inferred CD8^+^ T cells subsets. **G** Based on NEOITHS and NEO2IS, all included samples were segregated into four subgroups: C1-C4 that correspond to distinct TMEs. **H** CIBERSORTx-estimated immune cell fractions within the defined immune subtypes. **I** Three immune subgroups were differentiated by distinct clinical response to ICBs in NSCLCs and melanomas. **J** Kaplan–Meier plots for relapse-free (left panel) and overall survival (right panel) from ICB cohorts stratified by three defined immune subtypes.

To inquire into impact of intratumor heterogeneity (ITH) on immunotherapy response and more resistance mechanisms, we subsequently evaluated the neoantigen ITH score (NEOITHS) for each tumor. Given that CD8^+^ T cell comprises multiple interconnected subpopulations, we examined CIBERSORTx fractions of four major CD8^+^ T cell subclusters from scRNA-seq datasets (Fig. 5A) and all bulk RNA-seq data (Fig. S5a). We further linked these scores to the heterogeneity within the CD8^+^ TIL lineage caught in an in vivo détente against the progressively growing tumor [15]. Surprisingly, more effector/memory cells and progenitor CD8^+^ Tex were identified in TCGA tumors with higher NEOITHS, while terminally exhausted cell population abundance showed in opposite direction (Fig. 5B). Furthermore, increased enrichment of terminally exhausted-like TILs was detected in NSCLC and melanoma samples with lower NEOITHS in validation datasets, whereas tumors with more heterogeneous neoantigens were enriched with activated TILs, effector/memory CD8^+^ T cell and progenitor CD8^+^ Tex (Fig. 5C). Unlike aforementioned NEO2IS, we observed heightened expression of several immune-checkpoint molecules in tumors with lower NEOITHS (Fig. 5D). Moreover, this score was also associated with better prognosis of NSCLC patients receiving ICB treatments (Fig. 5E). Although no association of this score and TCR diversity with relapse-free survivals was observed, NEOITHS predicted overall survivals in melanoma patients (Fig. 5E). To better understand the broader implication of evolution dynamics of neoantigens and neoantigen-T cell interactions, we analyzed the TCR repertoire of T cells in tumors from RNA-seq data and sought to determine the relationship between distinct TIL populations and TCR diversity measurements. Intriguingly, clonotypic diversity estimated by normalized Shannon index was positively associated with effector/memory TILs or progenitor CD8^+^ Tex while inversely correlated with terminally CD8^+^ Tex (Fig. 5F). These data suggest that NEOITHS can delineate infiltration degree of CD8^+^ T-cell with different differentiation states, and this metric might also reflect the diverse TCR repertoires recognizing neoantigens(Fig. 5F).

Subsequently, we explored how persistent load and the heterogeneity of neoantigens influenced on immunomodulatory sensitivity. Given above-mentioned observations that T cell-inflamed gene signatures and immune-escape were associated with a higher likelihood of high NEO2IS presence, we defined four categories of neoantigen-T cell interactions under heterogeneous immune-mediated negative selection pressures (Fig. 5G), namely, C1 (high NEO2IS & high NEOITH), C2 (high NEO2IS & low NEOITHS), C3 (low NEO2IS low & high NEOITHS) and C4 (low NEO2IS & low NEOITH). The corresponding T cell states and TME characteristics of C1-C4 tumors also varied substantially, as inferred from their distinct cell subpopulations (Fig. 5H). C1 and C2 with chronic antigenic exposure were both the infiltrated-inflamed type (so-called ‘hot’ tumors) as their TMEs were enriched in CTLs. C2 tumors (highly immunosuppressive, high ISP) displayed a more prominent terminally CD8^+^ Tex phenotype, while C1 (low ISP) was observed with a relatively lower abundance of Tregs in view of heterogenous neoantigen signals. C3 and C4 were both immunologically ‘cold’ tumors with low NEO2IS. C3 (infiltrated-excluded and high ISP) exhibited the highest fractions of suppressive M2 macrophages and Tregs. Whereas C4 tumors (immune-desert) with a dormancy phenotype had a relatively lower levels of HLA/B2M gene expression, NKT and dendritic cell infiltration than C3. We next determined whether distinct immune subtypes exhibited differences in treatment response driven by the mechanism of reversing T-cell exhaustion. When combined two high ISP subtypes (C2 and C3) together, a significant difference of clinical benefit to ICBs and prognosis was seen between C1, C2/C3, and C4 tumors (Fig. 5I). As expected, C4 had the poorest outcomes to immunotherapy owing to defects in the molecular machinery for antigen presentation. High ISP groups exhibited resistance to anti-PD1 inhibitors and poor prognosis in terms of enrichment for immunosuppressive cells and terminally exhausted CD8^+^ T cells, indicating that these dysfunctional immune subpopulations in the TME may contribute to ICB refractoriness. Tumor clonal architecture is sculpted by immunoediting. C1 tumors with both high NEO2IS and NEOITHS were deemed as immune-escaped and to experience stronger immune-mediated negative selection pressures (effective immune surveillance) [21]. Besides, when compared with ISP tumors, C1 was enriched with a larger proportion of progenitor CD8^+^ T cells and lower fractions of terminally CD8^+^ Tex. Therefore, C1 is most likely to reinvigorate exhausted T cells and has the most favorable prognosis after receiving ICBs (Fig. 5J).

## Discussion

Extensive studies have revealed that SNV and indel neoantigen load strongly correlates with clinical response to ICB therapy. Recent researches indicate that gene fusion is an important source of tumor-specific antigens that can elicit a cytotoxic T-cell response [10]. Therefore, future clinical trials of personalized treatments and industrial manufacturing processes should now be designed to allow analysis of patients’ T-cell responses to all possible types of antigens and utilize them as immunotherapeutic targets [22]. In this study, we comprehensively analyzed of WES and RNA-seq data to provide a detailed landscape of SNV, indel and fusion-derived neoantigens presented by MHC I molecules in tumors. Our analysis showed that exhausted CD8^+^ T-cell markers were associated with predicted neoantigen load scores after adjusting clinicopathologic covariates. Furthermore, a NEO2IS was developed to evaluate the immunogenic potency of candidate neoantigens and predict clinical efficacy of 5 ICB cohorts. We found that our defined NEO2IS improved discrimination of responses to immunotherapy in lung cancer and melanoma patients. We reckon that our work would aid clinicians in making treatment decisions and conducting personalized therapies. Intriguingly, TMB failed to show significant explanatory power on survival or clinical benefit of Abbott patients [23]. This is in part due to the ignorance of fusion neoantigens in calculation of TMB. Moreover, fusion-derived NLS was significantly higher in responders than non-responders, while no significant association of SNV or indel-derived NLS with clinical benefit was detected in the 51 melanomas. A previous study showed that fusion neoantigens had the highest immunogenic potential in 32.2% of TCGA patients, especially for patients with low SNV and indel burdens [24]. Although the overall neoantigen load of fusion genes was found to be substantially lower than the total SNV-neoantigen burden across different types of solid tumors [25] (i.e., lung cancer and melanomas in this study, Fig S1, S3), some fusion neopeptides are likely to induce a stronger immunogenic microenvironment and antitumor immunity. In a study of an exceptional ICB responder with head and neck cancer, circulating CD8^+^ T cells were proved to recognize a peptide derived from a novel DEK-AFF2 gene fusion. In this study, we observed neoantigens from a highly recurrent fusion gene in lung cancer showing strong immunogenic potentials (Fig. S5b), suggesting that EML4-ALK derived LAFSGIMIVY peptide may stimulate significant CD8^+^ TIL responses and become an valuable immunotherapeutic target for vaccine or engineered T cell therapies [26].

We further investigated the potential molecular mechanisms accounting for our NEO2IS model that improved the accuracy of predictions of patient responses to immunotherapy. Previous studies have reported that gene deficiencies in two DDR pathways of MMR and BER (POLE) led to a durable clinical benefit from immunotherapies [16, 27]. Co-mutations in multiple DDR pathways were revealed to associate with higher genomic instability, higher neoantigen burden and TMB; therefore, DDR genomic signatures could serve as potential prognostic biomarkers for ICBs [16]. Our analyses showed that TCGA tumors with activated DDR pathways manifested an immune phenotypic profile of increased NEO2IS. Consistent with aforementioned results involving DDR pathways in those tumors, the higher their NEO2IS identified, the higher their ploidy exhibited. This suggested that persistence of clones inside tumors needs perturbing the DNA mismatch repair machinery to induce genomic instability of tumor cells, leading to an increased burden of neoantigens. This circumstance of aneuploidy also reflected an absence of immunoediting and acquisition of tumor escape mechanisms [11]. Chromosomal instability can also be deemed as a later event after the tumor cells have been loaded with neoantigens based on the immunoediting theory [11]. The aberrant state (genomic instability) of validation samples was also reflected by NEO2IS, suggesting that the DNA mismatch repair-deficient tumors are more likely to generate and accumulate neoantigens with immunogenic potentials (recognized by the immune system) and have higher NEO2IS. Additionally, tumors with high NEO2IS expressed high levels of T-effector and IFNγ-associated genes as well as multiple immune-checkpoint molecules, such as CD274, PDCD1, CTLA4, LAG3 and TNFRSF8, suggesting the existence of ongoing immune activity but functionally suppressed immune response in these tumors. PDL1 expression is induced by IFNγ (secreted by activated NK and T cells), and is abundant in carcinomas and TME [28]. In the clinic, PDL1 levels in diagnostic biopsies are commonly employed to predict ICI sensitivity in patients with various tumors [29] (i.e., Rizvi LUAD patients, Fig. S5c). However, due to non-standardized criteria and cut-offs for assessing positivity, PDL1 expression is reported as an imperfect biomarker of ICB response by contradictory results of multiple studies. A fraction of PDL1^+^ tumors fails to respond to ICB and durable responses are observed in PDL1^−^ tumors [30, 31]. That’s caused by an important mechanism of immune escape involving tumor cells’ defects in the IFNγ receptor kinases JAK1 and JAK2 and the signal transducer and activator of transcription (STAT) molecules [18, 19]. While PDL1 level seems to be a relevant prognostic biomarker to rationalize the effective pembrolizumab treatment, it may merely reflect tumor inflammation status and indicate an overall immune system status. Thus, it should not be treated as a predictor of immunotherapy efficacy mechanistically. Instead, a combination of PDL1 expression with other indicators, such as abundant TIL infiltration and neoantigen burden, may offer better predictiveness. Continuous interferon-gamma exposure can lead to immunoediting of cancer cells, resulting in immune escape [32]. Our analysis showed that NEO2IS correlated significantly with PDL1 and STAT3 pY705 abundance (Fig. S5d), indicating that tumor cells with more immunogenic neoantigens required an immune-suppressive microenvironment (i.e., high PDL1 expression, deregulation of molecules involved in IFN-γ signaling pathway) to survive immune cell attacks. Another feature of tumors linked to immune evasion is LOHHLA which was found to associate with higher NEO2IS. NEO2IS can also reflect clinical features (tumor size and metastasis), implying progressive T cell dysfunction at advanced stage of tumors and terminal differentiation of CD8^+^ Tex under constantly elevated antigenic exposure in tumors. While the determinants of TME are complex and multifactorial, our composite NEO2IS helps depict immunoreactivity, infiltration and exhaustion degree of CD8^+^ T lymphocytes in responses to peptides derived from all possible classes of somatic mutations.

Adaptive immunity is operational only in tumor regions displaying an evolving neoantigen landscape, pointing to clonal evolution dictated by immune cells (immunoediting). Moreover, most CD8^+^ CTLs display heterogeneous and limited reactivity against neoantigens; therefore, it’s important to keep the migratory capacity of specific immune subsets [33] and the replenishment of tumor-infiltrating immune cells from the circulation or adjacent normal tissues [34] for the interpretation of immunological ITH along both the spatial and temporal dimensions.

We investigated the impact of clonality on neoantigen recognition in tumors with diverse clonal composition by using our defined NEOITHS. To assess whether there was consistency between neoantigen diversity and the heterogeneity within the CD8^+^ TIL lineage, we analyzed a refined clustering of CD8^+^ T cells and their clonotypic diversity. Surprisingly, we observed a higher abundance of terminally exhausted-like TILs in both NSCLCs and melanomas with lower NEOITHS, while enriched activated TILs, effector/memory CD8^+^ T cell and progenitor CD8^+^ Tex in tumor with more heterogeneous neoantigens. In a previous study, ccRCC in metastatic disease was observed with an enrichment of terminally exhausted CD8^+^ T cells and this subpopulation was restricted in TCR diversity [20]. Indeed, in our study, the entropy index used to estimate TCR diversity showed negative association with terminally CD8^+^ Tex fractions while positively linked to abundance of effector/memory TILs or progenitor CD8^+^ Tex. The degree of clonal expansion in a T-cell population reflected by TCR repertoire diversity was consistent with the neoantigen heterogeneity under evolutionary selections. Therefore, our NEOITHS metric can be considered as an indicator of negative selection pressures against the predicted neoantigens from the immune surveillance. Higher NEOITHS means the presence of substantial clonal expansion of effector/memory CD8^+^ T cells and progenitor CD8^+^ Tex after priming, manifesting stronger selective pressures and reflecting numerous pre-existing subclones that evaded detection by the immune system. However, if low NEOITHS was detected in tumors, a large subpopulation of CD8^+^ TILs maybe reactive against neoantigens but terminally dysfunctional CD8^+^ Tex expressing high levels of PD-1 and accompanied by heightened co-inhibitory receptor expression (including CTLA4, HAVCR2, LAG3, CD160, and TIGIT).

Multiple studies have reported that high TCR repertoire diversity associates with improved survival in multiple tumors, and response to CTLA-4 inhibition in melanoma and hepatocellular carcinoma [35–37]. However, other studies have observed that low TCR repertoire diversity (high clonality) correlates with clinical response to PD-1 axis inhibition in melanoma and urothelial carcinoma [38, 39]. Shannon entropy was recommended to estimate TCR repertoires derived from RNA-seq datasets; while the use of evenness and “productive clonality” (1-evenness) is strongly discouraged [40]. Intriguingly, in our study, both high NEOITHS and TCR diversity associated with improved survival in external NSCLC ICB cohort. However, NEOITHS can predict OS of melanoma patients, while only higher TCR clonality(1-nomalized Shannon entropy) correlates with relapse-free survival of Abbott melanoma patients (Fig. S5e). These results highlight the complexity of TCR repertoire biology, along with the importance of interpretating spatiotemporal immunological ITH by integrating diversity of both neoantigens and T cell repertoires and by keeping neoantigen-T cell interactions under careful consideration [40]. We consequently introduced four categories of immune subtypes by combining metrics of NEO2IS with NEOITHS, namely, C1-C4, corresponding to different TMEs. In the infiltrated-inflamed type (high NEO2IS), upon a sufficiently strong neoantigen signal, the immune cells could be recruited and manifested strong cytolytic activity (the ultimate effector mechanism in the cancer immunity cycle, Fig. S5a). Given the heterogeneity and plasticity of the tumor ecosystem, a subpopulation of these tumor cells probably exploited immune-evasive TME signaling pathways to enable escape from the immune system or treatments. Therefore, this type was further divided into C1 (high NEOITHS, low ISP) and C2 (low NEOITHS, high ISP) subsets. They were characterized by two phenotypically and transcriptionally distinct subpopulations of exhausted CD8^+^ T cells (progenitor and terminally CD8^+^ Tex, respectively). Among ‘cold’ tumors with low NEO2IS confined to the periphery of the TME, C3 (high NEOITHS) exhibited the highest fractions of suppressive M2 macrophages and Tregs, thus was defined as a infiltrated-excluded type with high ISP. C4 tumors (low NEOITHS) are prone to have defects associated with infiltration of APCs into the tumor tissue and thus categorized as an immune-desert subtype with a dormancy state, in which antigen presentation and priming of an adaptive immune response were more likely inefficient. C4 showed minimal clinical benefit from ICBs and the worst prognosis, given the tumor cell-intrinsic mechanisms that lead to primary/adaptive resistance to immunotherapy. A previous study proposed an immune dysfunction circuit constituted by inhibitory interactions between terminally exhausted CD8^+^ T cells and M2-like macrophages. By suppressing antitumor-immune activity, this immune circuit may lead to a worse prognosis [20]. Due to stronger immunosuppressive signals in the TME or tumor cell-extrinsic factors that reduced possibilities of reinvigorating exhausted T cells, the high ISP group (C2 and C3) with weakened immunity was less likely to respond to anti-immunosuppressive strategies. In contrast, the ICB treatments for C1 tumors were effective and predisposed to overcome tumor-induced immune suppression. This is consistent with previous findings that progenitor exhausted TILs can respond to anti-PD-1 therapy, but terminally exhausted TILs cannot; and melanoma patients who have a higher percentage of progenitor exhausted cells experience a longer duration of response to checkpoint-blockade therapy [41]. This subclass presents a potential geographical feature established to recruit and activate adaptive immune cells, for instance, tertiary lymphoid structures (TLSs) that act as key sites for the initiation of anticancer immunity and often correlates with a positive prognosis [42]. As was revealed by the evolutionary dynamics of negatively selected neoantigens in growing tumors [21], the complexity of the evolving tumor-immune interplays may contribute to the emergence of immunological ITH and distinct sensitivity to ICB treatments.

Even though our work provides a comprehensive overview of neoantigens originated from all possible somatic mutations in NSCLC and melanomas, there are still a few limitations. First, we focused solely on 8–11 kmer neopeptides displayed by MHC I without considering 13-15 kmer peptides presented by MHC II, which could also be potential neoantigens and elicit a cytotoxic T cell response [43]. Second, neoantigen ITH information alone does not completely recapitulate the full scale of molecular ITH in these samples. Lastly, the immunogenicity of predicted neoepitopes remains to be further validated in assays with the autologous T cells.

As our results suggest, the NEO2IS has a superior predictive power over TMB for clinical efficacy of ICB therapy. TCR repertoire diversity is consistent with the neoantigen heterogeneity under evolutionary selections. The NEOITHS reflects the heterogeneity within CD8+ Tex lineage, delineates infiltration degree of CD8^+^ TIL with different differentiation states and manifests distinct selective pressures in the TME. Our findings offer tremendous insight into molecular determinants underlying cancer immunotherapy and provide an opportunity for the development of neoantigen-based therapeutic vaccines and T-cell therapies targeting multiple clonal neoantigens. Our defined immune subtypes can be used for predicting immunotherapy response and overall prognosis.

## Materials and methods

### Clinical efficacy evaluation

In five included immunotherapy cohorts, lung cancer patients were treated with pembrolizumab (anti-PD-1) and melanoma patients were treated with nivolumab, ipilimumab, tremelimumab or pembrolizumab (anti-CTLA4 or anti-PD-1 therapy). ICB response was assessed by using Response Evaluation Criteria in Solid Tumors (RECIST) version 1.1 after treatments. To classify treatment response, a durable clinical benefit (DCB) was defined using complete response (CR) or partial response (PR) or stable disease (SD) for more than 6 months. No durable benefit (NDB) was defined as progressive disease (PD) or a stable disease lasting 6 months or less. The NSCLC and melanoma patients with known ICB response outcomes were categorized as responders (CR or PR or DCB) and non-responders (SD or PD or NDB).

### HLA calling and neoantigen predictions

Patient-specific HLA calls were determined from normal WES data by Polysolver [44], a standard HLA inference tool. Based on the translated protein FASTA sequences output by NeoPredPipe (parameter: --preponly) [45], all possible peptides (~11-amino acids in length) containing nonsynonymous mutations (for SNVs and indels) were retained. Fused regions encompassing the 13-amino acids in front and rear of the fusion breakpoint were used to describe the impact of the fusion event on the coding regions.

Next, mutated peptides derived from 3 different types of somatic alterations as well as corresponding patient-specific HLA calls were queried for peptide-MHC complex (pMHC) binding affinity using NetMHCpan 4.1 (https://services.healthtech.dtu.dk/service.php?NetMHCpan-4.1) [46]. Strong binders with a %rank<0.5 were retained as the input of deepHLApan(version 1.1) [47]. According to outputs of deepHLApan model, a neoantigen score was calculated as the value of binding score × immunogenic score for each neoantigen. Then, the candidate neoantigens are screened out using the following criteria: (i) Predicted neoantigens with an immunogenic score >0.5. (ii) Neoantigens with lower five percent of neoantigen scores were excluded. (iii) SNV and indel neoantigens with an expression of TPM = 0 were removed from consideration. Finally, the number of all remaining neoantigens for sample i were summed as ni, and a neoantigen load score (NLS) was calculated as $NLSi \,=\, \mathop {\sum}\nolimits_1^{ni} {neoantigen\;score}$.

### Single-cell RNA-seq data and T cell receptor (TCR) analysis

For scRNA-seq data analysis, quality control was first applied to filter out low-quality cells or genes using the criteria of original publications. Next, we performed the standard Seurat V4 procedure (including NormalizeData, ScaleData and PCA) for GSE179994 and for GSE120575 without performing “NormalizeData”. The top 3000 and 4000 highly variable genes were used respectively for principal component analysis (PCA) of above two datasets. We ran Harmony on the top 50 PCs for batch effects corrections, and then UMAP or TSNE for dimensionality reduction using dimension parameter of 10 within the Seurat workflow. The batch-corrected PCs were used for Louvain clustering of cells. To identify different CD8^+^ T cell subclusters, all single-cells classified as CD8^+^ were further extracted in two datasets, respectively. The clustering process for CD8^+^ T cells followed the exact steps described above. Differentially expressed genes between two groups of clusters were identified using a two-sided Wilcoxon rank rum test with Benjamini-Hochberg (BH) correction. Based on a cell-type marker gene list of GSE120575 (reference dataset), all single-cells of GSE179994 were then annotated using preranked gene set enrichment analysis (GSEA) with the fgsea v1.20.0 R package [48]. Similarly, subclusters of CD8^+^ T cells in two datasets were annotated based on a list of Human Cell Markers from CellMarker database (http://biocc.hrbmu.edu.cn/CellMarker/) [49] and transcriptional signatures for progenitor and terminally exhausted CD8^+^ T cells (Table S1) [50].

For TCR analysis, we applied the TRUST4 tool to reconstruct TCRs and identify T cell clones from 3 bulk RNA-seq datasets given its good performance, higher sensitivity and shorter runtimes [51].

Diversity of inferred TCR repertoires was then calculated as the metric of Shannon Entropy with a natural logarithm. TCR diversity scores (Shannon Entropy, Evenness, and Richness) of TCGA samples were assessed through published results (mitcr_sampleStatistics_20160714.tsv, https://gdc.cancer.gov/about-data/publications/pancanatlas).

### Immune cell infiltration and tumor microenvironment analysis

Relative abundance of immune cells was estimated with gene expression profiles of included tumors using R package “xCell” [52]. To estimate the abundance of exhausted CD8^+^ T cells (Tex) and multiple interconnected subpopulations, CIBERSORTx signature matrixes were generated as reference matrixes [53] by using immune cell types from NSCLC and melanoma single-cell data, respectively. According to the resulting signature matrixes, CIBERSORTx deconvolution was performed on the bulk RNA-seq datasets (TCGA-LUNG, TCGA-SKCM, SMC, Amato and Abbott) with quantile normalization disabled and with the number of permutations set to 1000.

To further extract and integrate tumor-microenvironmental features, by using the prcomp R function, PCA was performed on (1) the estimated fraction of myeloid cells, (2) the estimated fraction of infiltrating CD4^+^ lymphocytes, (3) the abundance of CIBERCORTx CD8^+^ Tex populations, (4) the estimated fraction of infiltrating CD8+ lymphocytes and expression of two marker genes [54], and (5) expression of HLA/B2M gene [55], respectively. Principal component coordinates for each sample were extracted using the factoextra R package (https://github.com/kassambara/factoextra). Principal component 1 (PC1) of 5 PCA above was denoted as (1) TAMs to represent the overall abundance of tumor associated macrophages, (2) CD4-effector to represent the activation and helper role of CD4^+^ T cells, (3) cytolytic activity to simulate the cytolytic state of CD8^+^ T cells, (4) CD8-Tex to represent the overall exhaustion status of CD8^+^ T cell in tumors, and (5) meta-HLA to represent the overall HLA/B2M gene expression, respectively (Table S2).

### Linear regression modeling and Gene set enrichment analysis

Linear regression models were built using mRNA expression levels [log_2_(TPM + 1)] of TCGA NSCLC and SKCM samples as the response variable, and sex, age, tumor purity and predicted neoantigen load as predictors. Using the lm function in R software, the multivariate regression model with the following formula was fit.

$$mRNA\;expression\;of\;gene\;Y\,\sim\, \beta 1 \,\times\, sex \,+\, \beta 2 \,\times\, age \,+\, \beta 3 \,\times\, tumor\;purity \,+\, \beta 4 \,\times\, NLS$$

The correlation between 3 kinds of NLS and a set of immune genes was calculated respectively by using clusterProfiler R package [56] and performing Gene Set Enrichment Analysis (GSEA) on genes ranked by the t values from linear regression models [57]. Reference gene sets for REACTOME and KEGG pathways were derived from the Molecular Signature Database (MsigDB) (http://software.Broadinstitute.org/gsea/msigdb/index.jsp).

As is shown in Fig. 2D, to assess the role of tumor neoantigens derived from different mutational types to T-cell reaction (dominantly CD8^+^ T cell exhaustion), by using fusion-, SNV- and indel-derived NLS (X1~X3), HLA or B2M mutations(X4), expression level of CD80 (X5), abundance of Neutrophils (TAN), Cancer-Associated Fibroblasts (CAFs) and Tregs (X6~X8), TAMs and CD4-effector (X9~X10), sex, age and tumor purity (X11~X13) as predictors, our defined CD8-Tex (the response variable) was incorporated with 15 covariates into the following linear regression model:

$$\begin{array}{ll}CD8 \,\mbox{-}\, Tex \,\sim\, \beta 0 \,+\, \beta 1 \,\times\, fusion \,\mbox{-}\, NLS \,+\, \beta 2 \,\times\, SNV \,\mbox{-}\, NLS \,+\, \beta 3 \,\times\, indel \,\mbox{-}\, NLS \,+\, \beta 4\cr \\ \qquad\qquad\quad\,\times\, {{{\mathrm{HLA/B2M}}}}\;mutation \,+\, \beta 5 \,\times\, CD80\;expression \,+\, \beta 6 \,\times\, TAN \,+\, \beta 7 \,\times\, CAFs\cr \\ \qquad\qquad\quad\,+\, \beta 8 \times Tregs \,+\, \beta 9 \,\times\, TAMs \,+\, \beta 10 \,\times\, CD4 \,\mbox{-}\, effector \,+\, \beta 11 \,\times\, sex \,+\, \beta 12\cr \\ \qquad\qquad\quad\,\times\, age \,+\, \beta 13 \,\times\, tumor\;purity \,+\, \varepsilon \\ \end{array}$$

Before training, TCGA data were randomly split into a training and a testing portion (by 7 to 3). Using “caret” R package, ten-fold cross-validation was applied to evaluate model robustness on the training dataset internally. Likewise, SVR and gbm models were also trained using these features on two TCGA datasets. After feature selection with stepwise regression (both forward and backward selection), the final results of CD8-Tex model (Table S3) were used to yield our composite neoantigen load score (NEO2IS ~ β1 × fusion -NLS + β2 × SNV-NLS) to reflect the different states of neoantigen-based CD8^+^ T cell response (e.g., proliferation, cytotoxicity and exhaustion).

### Computation of neoantigen ITH score

Neoantigen ITH analysis was performed as follows. First, each predicted neoantigen was annotated with a cancer cell fraction (CCF) value. For neoantigens derived from SNVs or indels, CCF was calculated as follows [58]:

$${{{\mathrm{CCF}}}} \,=\, \frac{{VAF}}{{m \,\times\, purity}}(purity \,\times\, CN \,+\, 2(1 \,-\, purity))$$

where variant allele fraction (VAF) is the fraction of mutated reads for a given variant (estimated as the number of mutant reads spanning the position divided by the number of total reads of the position). CN and purity represent the copy number of the mutation’s genomic locus and the fraction of tumor cells in the sequenced sample, respectively. Purity was estimated using ABSOLUTE R package. Multiplicity of a mutation (m) is the number of DNA copies bearing a mutation m, which can be estimated from the VAF, purity and local copy number as ${{{\mathrm{m}}}} = {{{\mathrm{VAF}}}}/{{{\mathrm{purity}}}} \times ({{{\mathrm{purity}}}} \times {{{\mathrm{CN}}}} + 2(1 - {{{\mathrm{purity}}}}))$ [58]. In regions of clonal copy number, the multiplicity of a mutation is a strictly positive integer, so the most likely value can be obtained by rounding to the nearest non-zero integer: ${{{\mathrm{m}}}} \,=\, {{{\mathrm{max}}}}(1,{{{\mathrm{round}}}}({{{\mathrm{VAF}}}}/{{{\mathrm{purity}}}} \,\times\, ({{{\mathrm{purity}}}} \times {{{\mathrm{CN}}}} \,+\, 2(1 \,-\, {{{\mathrm{purity}}}}))))$, where round is a function that returns the nearest integer [58]. CCF values above 1 (arising from sequencing noise or copy-neutral loss-of-heterozygosity events) were assumed to be 1. For fusion neoantigens, we annotated neoantigens derived from onco/driver fusion genes with CCF = 1 and passenger genes with CCF = 0.5 since mutations in driver genes were prone to be clonal and early events compared to mutations in nondriver genes [59]. The list of oncogenes, tumor suppressor genes, protein kinase genes, and driver genes was obtained from a previously published result [25]. Then, each neoantigen from SNV or indel was considered as clonal neo if the CCF exceeded 0.84 and 0.9 (medians of CCF values for all melanomas and lung cancers) [60]. The neoantigen ITH score (NEOITHS), for sample i with the number of subclonal neo (N_s_) and the number of clonal neo (N_c_), was calculated as: $NEOITHS,\;i \,=\, Ns,\;i/(Ns,i \,+\, Nc,\;i)$. The tumor clones that had zero neoantigen were assigned a NEOITHS of 1.

### Statistical analysis

All statistical analyses were performed with R (v4.1.0). The number of NSMs in the coding region (38 Mb) for each tumor sample was used to compute and estimate the tumor mutation burden (TMB). Differentially expressed genes between two subgroups divided according to a given phenotype were identified by the limma package using a threshold of *p* value <0.1. Functional enrichment analysis was conducted using the clusterProfiler package. The significantly enriched signatures with q-value <0.3 were retained and then visualized by GOplot package. Gene set variation analysis (GSVA) was utilized for identifying pathways most related to the neoantigen model. GSVA was performed with a set of 186 KEGG pathway signatures by using the “GSVA” package [61]. Pathway signatures with adjusted *p* value <0.05 were considered significantly differentially enriched.

Heatmaps of predicted neoantigens were conducted and visualized by R package ‘ComplexHeatmap’. Wilcoxon rank sum test, Chi-squared (χ2) test and Fisher’s exact test were used for assessing associations of genomic, clinical and molecular features (i.e., mRNA and protein expression) with NEO2IS or defined immune subtypes, which were implemented and visualized by ggplot2 and ggpubr packages. We conducted survival analysis on all cohorts. Overall survival (OS) was defined as the date of treatment initiation to the date of death or last follow-up. Relapse-free survival (RFS) was defined as the time from treatment initiation to the disease progression or end of the current follow-up. Kaplan–Meier curve analyses and log-rank tests were performed by package ‘survminer’. Receiver operating characteristic (ROC) curve analyses were conducted using pROC package. For all statistical tests, two-tailed *P* < 0.05 denoted statistical significance, indicated by *P < 0.05; **P < 0.01; ***P < 0.001; ****P < 0.0001, and NS. denoted non-significance.

### Key Resources Table

▓

**Table:** 

Data resource	Source	Identifier
Deposited data		
TCGA level 4 RNA-seq, clinical and survival information (LUAD, LUSC and SKCM samples)	UCSC Xena database	https://gdc.xenahubs.net
TCGA level 3 somatic mutation data (LUAD, LUSC and SKCM samples)	Genomic Data Commons	https://portal.gdc.cancer.gov/
TCGA RPPA data (LUAD, LUSC and SKCM samples)	RPPA Core Facility, MD Anderson Cancer Center	http://app1.bioinformatics.mdanderson.org/tcpa/_design/basic/index.html
TCGA HLA allele information (LUAD, LUSC and SKCM samples)	The Cancer Immunome Atlas	https://tcia.at/home
TCGA fusion genes (LUAD, LUSC and SKCM samples)	ChimerDB 4.0 database	https://www.kobic.re.kr/chimerdb/
Raw WES data (Rizvi LUAD and Synder melanoma samples)	Database of Genotypes and Phenotypes	https://www.ncbi.nlm.nih.gov/projects/gap/cgi-bin/study.cgi?study_id=phs000980.v1.p1
		https://www.ncbi.nlm.nih.gov/projects/gap/cgi-bin/study.cgi?study_id=phs001041.v1.p1
Raw RNA-seq and WES data (Amato melanoma samples)	NCBI Sequence Read Archive database	https://trace.ncbi.nlm.nih.gov/Traces/study/?acc=SRP267584
		https://trace.ncbi.nlm.nih.gov/Traces/study/?acc=SRP217040
Raw and processed RNA-seq and WES data (Abbott melanoma samples)	Database of Genotypes and Phenotypes	https://www.ncbi.nlm.nih.gov/projects/gap/cgi-bin/study.cgi?study_id=phs002388.v1.p1
	NCBI Gene Expression Omnibus	https://www.ncbi.nlm.nih.gov/geo/query/acc.cgi?acc=GSE15996
Raw and processed RNA-seq and WES data (SMC NSCLC samples)	NCBI Sequence Read Archive database	https://trace.ncbi.nlm.nih.gov/Traces/study/?acc=SRP217040
	NCBI Gene Expression Omnibus	https://www.ncbi.nlm.nih.gov/geo/query/acc.cgi?acc=GSE203360
	European Genome-phenome Archive	https://ega-archive.org/datasets/EGAD00001005211
Processed single-cell RNA-seq data (NSCLC and melanoma samples)	NCBI Gene Expression Omnibus	https://www.ncbi.nlm.nih.gov/geo/query/acc.cgi?acc=GSE17794
		https://www.ncbi.nlm.nih.gov/geo/query/acc.cgi?acc=GSE120575
Software and algorithms		
R v4.1.0	The Comprehensive R Archive Network	https://www.r-project.org/
BWA v0.7.15		http://bio-bwa.sourceforge.net/
MuTect2 v4.1.0		https://github.com/broadinstitute/mutect
GATK v4.1.7.0	Van et al. [9]	https://github.com/gatk-workflows/gatk4-somatic-snvs-indels/
Polysolver	Shukla et al. [44]	https://github.com/jason-weirather/hla-polysolver
NetMHCpan v4.1	Reynisson et al. [46]	https://services.healthtech.dtu.dk/service.php?NetMHCpan-4.1
deepHLApan v1.1	Wu et al. [47]	https://github.com/jiujiezz/deephlapan
STAR v2.7.9a		https://github.com/alexdobin/STAR
STAR-Fusion v1.10.1	Haas et al. [62]	https://github.com/STAR-Fusion/STAR-Fusion
Arriba v2.2.1		https://github.com/suhrig/arriba
GeneFuse v0.6.1		https://github.com/OpenGene/genefuse
CNVkit v0.9.7		https://github.com/etal/cnvkit
TRUST4 v1.0.6	Song et al. [51]	https://github.com/liulab-dfci/TRUST4
Seurat v4.2.0	Hao et al. [63]	https://satijalab.org/seurat/
CIBERSORTx	Newman et al. [53]	https://cibersortx.stanford.edu/runcibersortx.php
ABSOLUTE v1.0.6		https://software.broadinstitute.org/cancer/cga/absolute
xCell v1.1.0	Aran et al. [52]	https://github.com/dviraran/xCell/
LOHHLA	McGranahan et al. [12]	https://bitbucket.org/mcgranahanlab/lohhla/src/master/
survminer v0.4.9	The CRAN package repository	https://cran.r-project.org/package=survminer
survival v3.2.11	The CRAN package repository	https://cran.r-project.org/package=survival
factoextra v1.0.7	The CRAN package repository	https://cran.r-project.org/package=factoextra
clusterProfiler v4.1.4	Yu et al. [56]	http://bioconductor.org/packages/release/bioc/html/clusterProfiler.html
fgsea v1.20.0	Sergushichev et al. [48]	http://bioconductor.org/packages/release/bioc/html/fgsea.html
GSVA v1.40.0	Hänzelmann et al. [61]	https://bioconductor.org/packages/release/bioc/html/GSVA.html

## Supplementary information

Supplementary Materials and Figures

Supplementary Table1

Supplementary Table2

Supplementary Table3

## Data Availability

All datasets analyzed in this study were published previously and publicly available. The corresponding descriptions for data collection and preprocessing steps are described in the Supplementary Materials. These accession numbers for the datasets are listed in the key resources table.

## Code availability

Codes used in this study are available at GitHub (https://github.com/RH-LUO/NEO2IS_ITHS).
