# Supplementary material for: A novel integrated approach to predicting cancer immunotherapy efficacy
Source: Oncogene. 2023 Apr 26;42(23):1913–25. doi: 10.1038/s41388-023-02670-1 (PMC10244162; doi:10.1038/s41388-023-02670-1)
Supplement: Supplementary file 1 — Supplementary Materials and Figures [file 41388_2023_2670_MOESM1_ESM.pdf]

# Supplementary Materials:

## 1. Dataset and preprocessing descriptions

### Training data collection

TCGA WES data (VCF files of MuTect2 variant calling pipeline) for lung squamous cell carcinoma (LUSC, n=489), lung adenocarcinoma (LUAD, n=511) and skin cutaneous melanoma samples (SKCM, n=469) were requested from the NCI GDC Repository (https://gdc.cancer.gov). Human Leukocyte Antigen (HLA) allele information corresponding to the LUAD, LUSC and SKCM samples was obtained from The Cancer Immunome Atlas (https://tcia.at/home) [1].

Processed single-cell RNA sequencing (scRNA-seq) data of lung cancer and melanoma were obtained from the Gene Expression Omnibus (GEO; accession number: GSE17994 and GSE120575). Bulk RNA-seq level 3 expression data (normalized read counts) of the 1019 NSCLC and 474 melanoma tissues were downloaded from https://tcga.xenahubs.net. The read count was further converted into Transcripts Per Kilobase Million (TPM) quantification. Fusion gene lists for LUAD (n=471), LUSC (n=485) and SKCM (n=451) samples were retrieved from ChimerDB 4.0 database (https://www.kobic.re.kr/chimerdb/) [2]. Location information of the fusion events aligned to the human reference genome (version 19, hg19) was further lifted to the hg38 genome.

To further assess protein levels of tumors, protein expression profiles (reverse phase protein array [RPPA] data) of TCGA cohort (including 362 LUAD, 325 LUSC and 354 SKCM samples) were extracted from MD Anderson (http://app1.bioinformatics.mdanderson.org/tcpa/_design/basic/index.html).

### Immunotherapy cohort data

Raw bulk RNA-seq data (n=27) of SMC NSCLC cohort were downloaded from Sequence Read Archive (NCBI SRA: SRP217040). Processed gene expression data for these 27 tumor samples were downloaded from GSE135222. Corresponding WES data (including FASTQ files and VCF files) and associated HLA allele data of this cohort (n=146) were requested from European Genome-phenome Archive (EGA) under accession number EGAD00001005211 and previous studies [3, 4]. The tumor and matched germline WES FASTQ files for another independent LUAD cohort (Rizivi, n=34) were obtained from the Database of Genotypes and Phenotypes (dbGaP) under accession no. phs000980.v1.p1. All above lung cancer patients were treated with pembrolizumab (anti-PD-1). In SMC cohort, ICB treatment response information for only 122 patients can be obtained.

WES data of three melanoma cohorts (Abbott cohort, n=51; Amato cohort, n=52; Snyder cohort, n=64) were obtained from dbGap: phs002388.v1.p1; SRA: SRP267584 and dbGap: phs001041.v1.p1. Matched bulk RNA-seq data for 47 melanomas from Abbott cohort and 12 from Amato cohort were available at dbGap: phs002388.v1.p1 and SRA: SRP250849. Corresponding RNA expression data were downloaded from the supplementary table of Abbott's publication [5] and GSE15996. All included melanoma patients were treated with nivolumab, ipilimumab, tremelimumab or pembrolizumab (anti-CTLA4 or anti-PD-1 therapy). Corresponding clinical information of five studies above was retrieved from related supplementary files in the original publications [6] and from GEO database.

### Raw sequence data processing, somatic mutation calling and annotation

Somatic variants and VCF files were called using raw data of five ICB cohorts. First, the quality control for FASTQ files was assessed by using Trimmomatic (version 0.39)[7] and Samtools (version 1.9)[8]. Then, the ILLUMINACLIP step (keepBothReads is set True) and Sliding window

trimming were performed. Leading and trailing low quality (quality reading below 5) or N bases were removed. The raw reads (FASTQ files) were mapped to the hg38 genome using BWA-men (version 0.7.15)[9] with parameters -t 8 -T 0. Local realignment around indels and base quality recalibration of BAM files were performed with Genome Analysis Toolkit (GATK, version 4.1.7.0) [10]. MuTect2 (version 4.1.0.0)[11] was next applied with default settings to detect SNVs and small indels in 34 pairs of tumors and matched normal samples. FilterMutectCalls was performed with default parameters on Mutect2 for selecting the most reliable variant calls.

Somatic variants of two TCGA and five ICB datasets were further annotated using ANNOVAR [12] . In addition, functional impact prediction of mutations was carried out by the Variant Effect Predictor (VEP) from Ensembl. For both somatic SNVs and indels, the minimum variant allelic fraction was set to 2%. Indels that were annotated in genome repeat regions were removed. Germline variants present in the dbSNP database and common variants with AF over 5% reported by the 1000 Genomes Project (1000G) and the Exome Aggregation Consortium (ExAC) database[13, 14] were further filtered out.

For 5 ICB cohorts, CNVkit (version 0.9.7)[15] was employed for inferring somatic copy number alterations (SCNAs) in the aligned sequence reads (sorted BAM files). ABSOLUTE (v1.0.6)[16] was applied for estimating tumor purity and ploidy of these samples based on their MAF files and the copy number data (output files of CNVkit). The loss of heterozygosity (LOH) in at the HLA allele was inferred from their WES data using the algorithm LOHHLA[17]. A loss of heterozygosity event was reported when the significance of allelic imbalance was reached (p-value <0.01), and when the estimated copy number using binning and B-allele frequency settings was lower than 0.5. For TCGA samples, their purity/ploidy file (ABSOLUTE, TCGA_mastercalls.abs_tables_JSedit.fixed.txt) was downloaded at https://gdc.cancer.gov/about-data/publications/pancanatlas. The LOH-HLA information of TCGA cases was obtained from Yang's publication[18].

### Fusion gene detection and filtering

STAR-Fusion (v1.10.1) and Arriba (v2.2.1) showed a higher sensitivity in detecting the fusion events and was both utilized in the GDC gene fusion pipeline. Therefore, the two tools were employed for detecting gene fusions from the RNA-seq data of SMC, Abbott and Amato samples. First, reads were aligned to the hg38 reference genome using STAR(v2.7.9a)[19] followed by fusion calling with the following parameters --examine_coding_effect; --FusionInspector inspect[20] in STAR-Fusion. Specifically, the inspect mode of FusionInspector (v2.6.0) which comes bundled with STAR-Fusion was used to inspect prediction results from STAR-Fusion and retain reliable fusion transcripts. The prediction output files provided fused gene names, junction read count, and information on the breakpoint, LargeAnchorSupport and coding region impacts. Fusions not supported by LargeAnchor reads were filtered out. Then, Arriba was also applied to call fusions in NSCLCs and melanomas given its short runtimes and utility in both basic cancer research and clinical translation[21]. The union of fusion genes detected by the above two algorithms was retained for further analysis.

Moreover, GeneFuse (version 0.6.1)[22] was used to detect fusions from paired tumor and normal DNA of 5 ICB cohorts using a gene list of experimentally verified cancer-related fusions from the COSMIC database as well as two fusion files containing respectively TCGA NSCLC and SKCM fusion genes from ChimerDB 4.0. All genes in the fusion gene list files above were based on hg38 reference assembly. Fusion genes that were observed in match normal samples of two cohorts were further filtered out. Finally, for five ICB cohorts, the fusion results predicted from above three tools were filtered using FusionGDB 2.0 database[23].

## 2. Supporting results for main Fig. 1

The top-ranked frequent neoantigens predicted to strongly bind to patient's MHC class I molecules are shown in Fig. S1. In total, there were 4927 candidate neoantigens derived from fusion genes predicted across 811 TCGA lung cancer (TCGA-LUNG) samples. Fusion-associated neoantigens occurred most frequently in the following fusion genes: GOLGA3-RPS11, BMS1P20-IGLL5 and CLTC-VMP1 (Fig. S1a). In 1002 and 783 NSCLC samples, a total of 128848 SNVs-derived and 10625 indels-derived neopeptides were predicted to bind to MHC-I molecules, respectively. The most frequent peptides were induced by hotspot mutations in NSCLCs, including $EGFR^{L858R}$, $PIK3CA^{E545K,E542K}$, $KRAS^{G12V,G12A,G12D}$ and $TP53^{R158L}$ (Fig. S1a). In 469 TCGA melanoma (TCGA-SKCM) samples, there were 125770, 1554 and 2121 neoantigens originated from SNVs, indels and fusions, respectively. The predicted neoantigens occurred most frequently in $BRAF^{V600M}$, $NRAS^{Q61K}$, $RAC1^{P29S}$, TRO-MAGED2 and TTYH3-MAD1L1(Fig. S1a).

## 3. Supporting results for main Fig. 3

Neoantigens were highly sparse and infrequently shared between ICB cohort patients. In contrast to TCGA data, few neoantigens were derived from hotpot mutations like TP53, KRAS or BRAF except for $EGFR^{L858R}$ and $CDH6^{S524L}$ (Fig. S3a). Besides, the most frequent neoantigens were generated by fusion genes such as MAML3-ATXN3, GRM5-PIGK and CTSC–RAB38 in the combined NSCLC ICB cohort (SMC and Rizvi), and CTSC-RAB38, KANSL1-ARL17A and STEAP1B-RAPGEF5 in the combined melanoma ICB cohort (Synder, Amato and Abbott) (Fig. S3a).

# 4. Supplementary figures

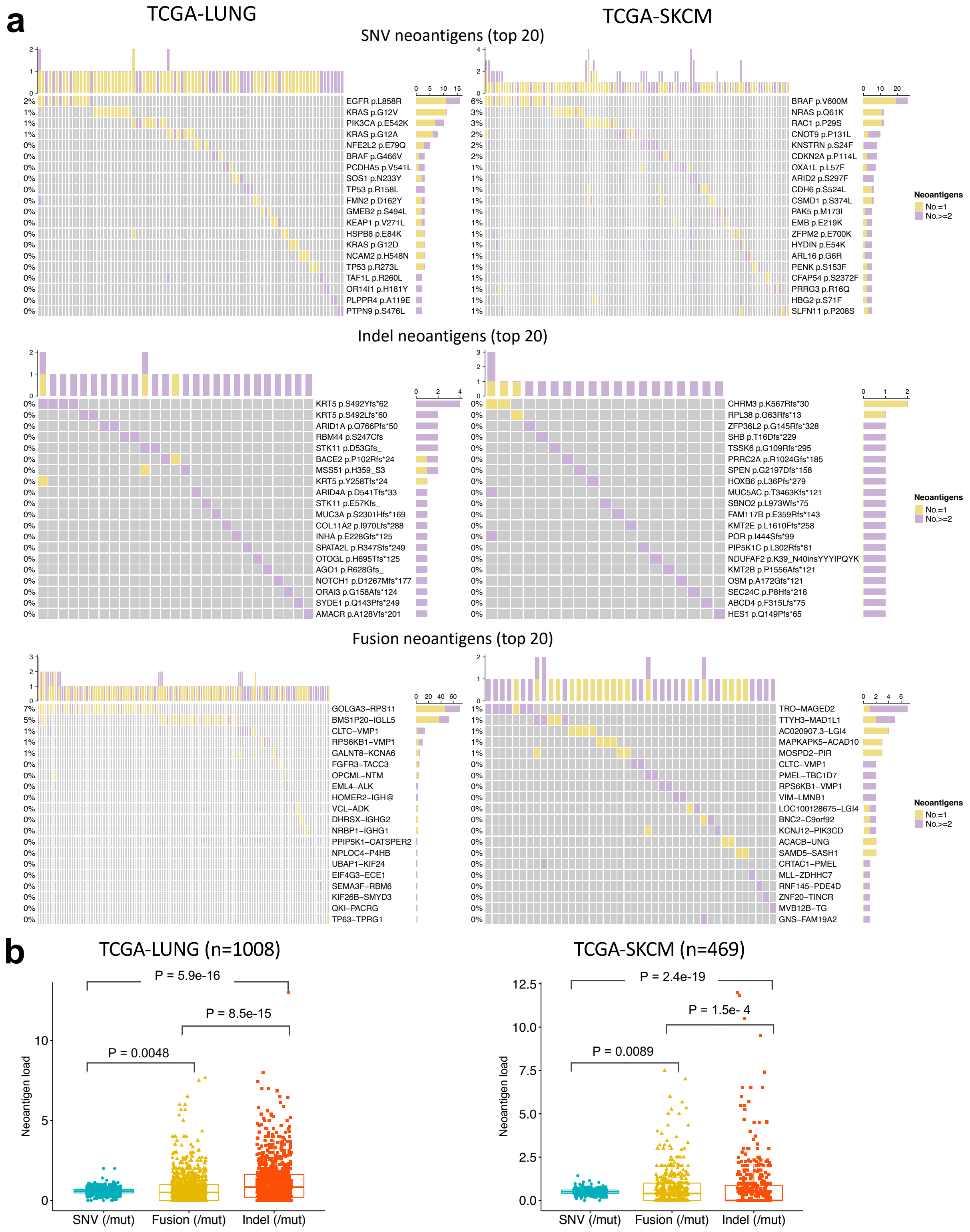


**Supplementary Fig. 1. a,** The landscape of neoantigens derived from SNVs (top), indels (middle) and fusion genes (bottom) in TCGA-LUNG (left) and TCGA-SKCM (right). **b,** More neoantigens are observed per indel than a SNV or a fusion gene in TCGA-LUNG (left panel); in contrast, neoantigens derived from each of SNVs were found to be more than those from each of fusions and indels in TCGA-SKCM (right panel). P values are derived from Wilcoxon test and adjusted for multiple testing with the Benjamini-Hochberg procedure.

# Fig. S2

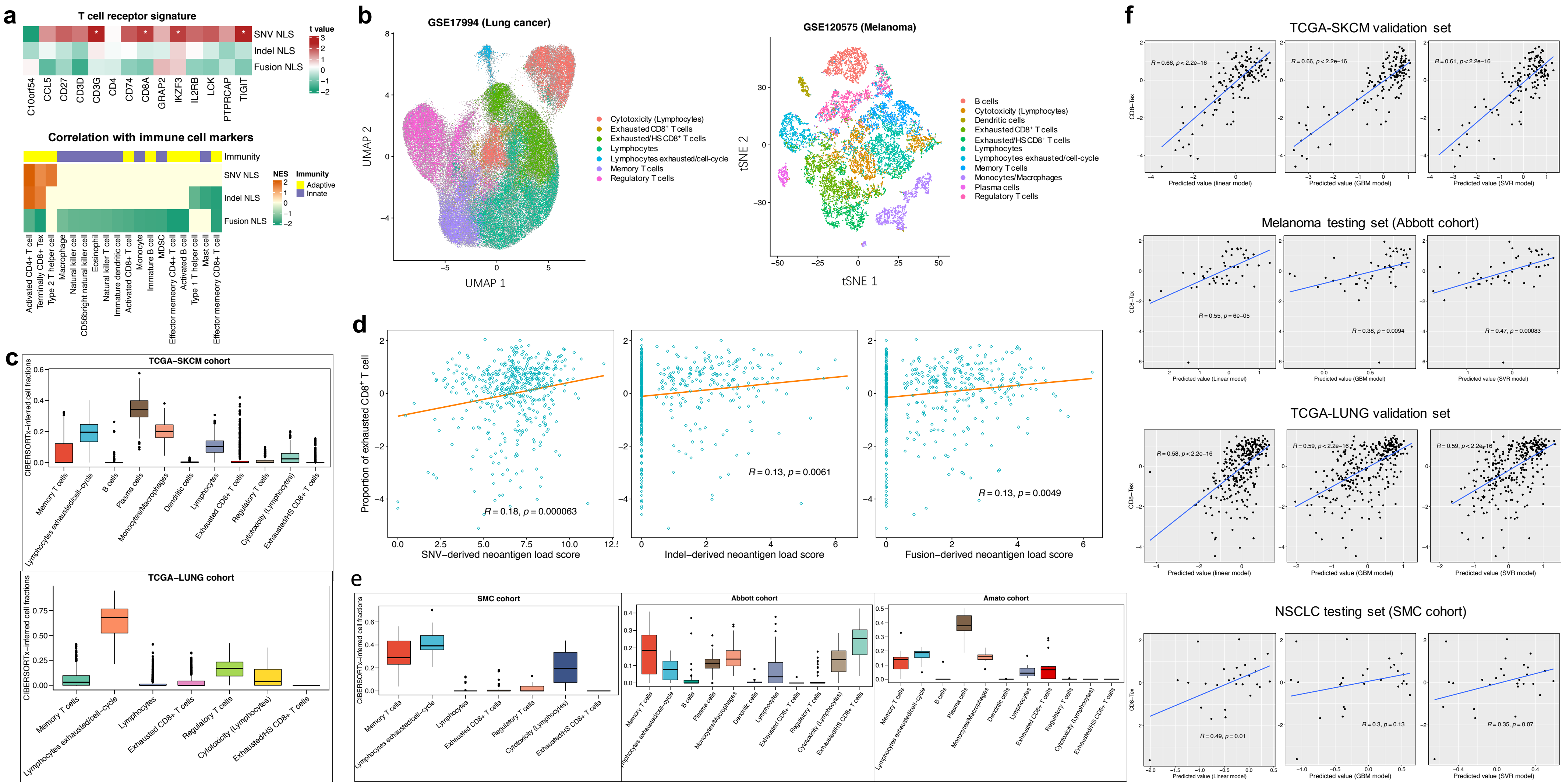


**Supplementary Fig. 2. a,** Heatmaps of correlations between mRNA expression levels of TCR gene signatures with NLS of three types (top panel), and GSEA normalized enrichment scores (NESs) for immune cell marker genes (bottom panel) in TCGA-SKCM primary samples. **b,** UMAP and tSNE plots of lymphoid and myeloid cells in scRNA-seq datasets of lung cancer and melanoma, colored by broad cell type. **c,** The cell-type fractions inferred using CIBERSORTx in bulk RNA-seq of TCGA-LUNG and TCGA-SKCM samples. **d,** The correlations of SNV-, indel- and fusion-derived NLS with CIBERSORTx-inferred exhausted CD8$^{+}$ T cells in TCGA-SKCM samples. **e,** The cell-type fractions in bulk RNA-seq of ICB samples inferred by CIBERSORTx. **f,** The performance comparison of three models in validation and testing datasets.

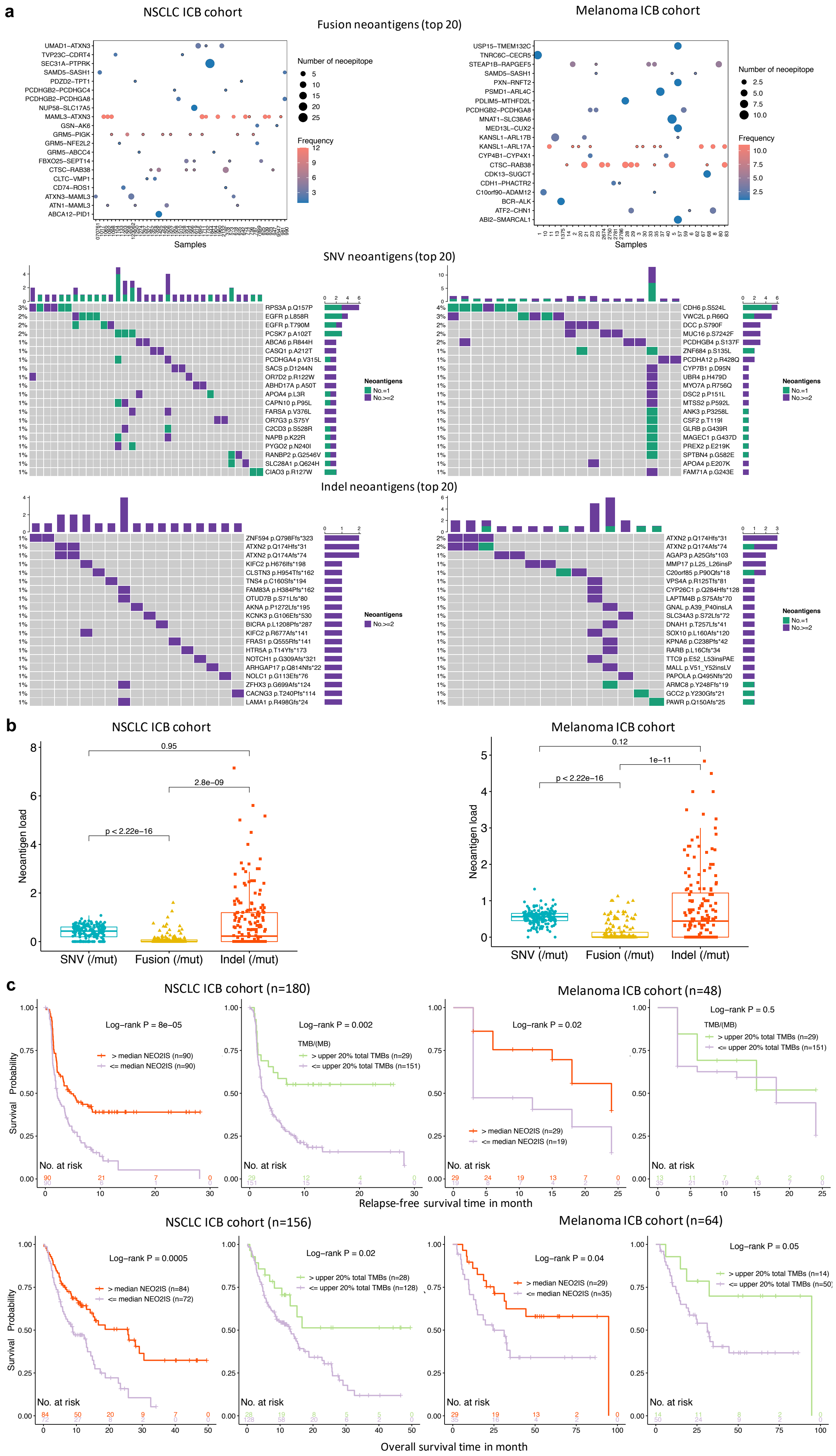
a
NSCLC ICB cohort
Melanoma ICB cohort
Fusion neoantigens (top 20)
UMAD1–ATXN3
TVP23C–CDRT4
SEC31A–PTPRK
SAMD5–SASH1
PDZD2–TPT1
PCDHGB2–PCDHGC4
PCDHGB2–PCDHGA8
NUP58–SLC17A5
MAML3–ATXN3
GSN–AK6
GRM5–PIGK
GRM5–NFE2L2
GRM5–ABCC4
FBXO25–SEPT14
CTSC–RAB38
CLTC–VMP1
CD74–ROS1
ATXN3–MAML3
ATN1–MAML3
ABCA12–PID1
Number of neoepitope
5
10
15
20
25
Frequency
12
9
6
3
Samples
USP15–TMEM132C
TNRC6C–CECR5
STEAP1B–RAPGEF5
SAMD5–SASH1
PXN–RNFT2
PSMD1–ARL4C
PDLIM5–MTHFD2L
PCDHGB2–PCDHGA8
MNAT1–SLC38A6
MED13L–CUX2
KANSL1–ARL17B
KANSL1–ARL17A
CYP4B1–CYP4X1
CTSC–RAB38
CDK13–SUGCT
CDH1–PHACTR2
C10orf90–ADAM12
BCR–ALK
ATF2–CHN1
ABI2–SMARCAL1
Number of neoepitope
2.5
5.0
7.5
10.0
Frequency
10.0
7.5
5.0
2.5
Samples
SNV neoantigens (top 20)
RPS3A p.Q157P
EGFR p.L858R
EGFR p.T790M
PCSK7 p.A102T
ABCA6 p.R844H
CASQ1 p.A212T
PCDHGA4 p.V315L
SACS p.D1244N
OR7D2 p.R122W
ABHD17A p.A50T
APOA4 p.L3R
CAPN10 p.P95L
FARSA p.V376L
OR7G3 p.S75Y
C2CD3 p.S528R
NAPB p.K22R
PYGO2 p.N240I
RANBP2 p.G2546V
SLC28A1 p.Q624H
CIAO3 p.R127W
Neoantigens
No.=1
No.>=2
CDH6 p.S524L
VWC2L p.R66Q
DCC p.S790F
MUC16 p.S7242F
PCDHGB4 p.S137F
ZNF684 p.S135L
PCDHA12 p.R428Q
CYP7B1 p.D95N
UBR4 p.H479D
MYO7A p.R756Q
DSC2 p.P151L
MTSS2 p.P592L
ANK3 p.P3258L
CSF2 p.T119I
GLRB p.G439R
MAGEC1 p.G437D
PREX2 p.E219K
SPTBN4 p.G582E
APOA4 p.E207K
FAM71A p.G243E
Neoantigens
No.=1
No.>=2
Indel neoantigens (top 20)
ZNF594 p.Q798Ffs*323
ATXN2 p.Q174Hfs*31
ATXN2 p.Q174Afs*74
KIFC2 p.H676Ifs*198
CLSTN3 p.H954Tfs*162
TNS4 p.C160Sfs*194
FAM83A p.H384Pfs*162
OTUD7B p.S71Lfs*80
AKNA p.P1272Lfs*195
KCNK3 p.G106Efs*530
BICRA p.L1208Pfs*287
KIFC2 p.R677Afs*141
FRAS1 p.Q555Rfs*141
HTR5A p.T14Yfs*173
NOTCH1 p.G309Afs*321
ARHGAP17 p.Q814Nfs*22
NOLC1 p.G113Efs*76
ZFHX3 p.G699Afs*124
CACNG3 p.T240Pfs*114
LAMA1 p.R498Gfs*24
Neoantigens
No.>=2
ATXN2 p.Q174Hfs*31
ATXN2 p.Q174Afs*74
AGAP3 p.A25Gfs*103
MMP17 p.L25_L26insP
C20orf85 p.P90Qfs*18
VPS4A p.R125Tfs*81
CYP26C1 p.Q284Hfs*128
LAPTM4B p.S75Afs*70
GNAL p.A39_P40insLA
SLC34A3 p.S72Lfs*72
DNAH1 p.T257Lfs*41
SOX10 p.L160Afs*120
KPNA6 p.C238Pfs*42
RARB p.L16Cfs*34
TTC9 p.E52_L53insPAE
MALL p.V51_Y52insLV
PAPOLA p.Q495Nfs*20
ARMC8 p.Y248Ffs*19
GCC2 p.Y230Gfs*21
PAWR p.Q150Afs*25
Neoantigens
No.=1
No.>=2
b
NSCLC ICB cohort
Neoantigen load
0.95
2.8e–09
p < 2.22e–16
SNV (/mut)
Fusion (/mut)
Indel (/mut)
Melanoma ICB cohort
Neoantigen load
0.12
1e–11
p < 2.22e–16
SNV (/mut)
Fusion (/mut)
Indel (/mut)
c
NSCLC ICB cohort (n=180)
Melanoma ICB cohort (n=48)
Survival Probability
Log–rank P = 8e–05
> median NEO2IS (n=90)
<= median NEO2IS (n=90)
No. at risk
Log–rank P = 0.002
TMB/(MB)
> upper 20% total TMBs (n=29)
<= upper 20% total TMBs (n=151)
No. at risk
Log–rank P = 0.02
> median NEO2IS (n=29)
<= median NEO2IS (n=19)
No. at risk
Log–rank P = 0.5
TMB/(MB)
> upper 20% total TMBs (n=29)
<= upper 20% total TMBs (n=151)
No. at risk
Relapse-free survival time in month
NSCLC ICB cohort (n=156)
Melanoma ICB cohort (n=64)
Survival Probability
Log–rank P = 0.0005
> median NEO2IS (n=84)
<= median NEO2IS (n=72)
No. at risk
Log–rank P = 0.02
> upper 20% total TMBs (n=28)
<= upper 20% total TMBs (n=128)
No. at risk
Log–rank P = 0.04
> median NEO2IS (n=29)
<= median NEO2IS (n=35)
No. at risk
Log–rank P = 0.05
> upper 20% total TMBs (n=14)
<= upper 20% total TMBs (n=50)
No. at risk
Overall survival time in month

**Supplementary Fig. 3.** Neoantigens inferred from ICB cohorts and clinical efficacy evaluated by the NEO2IS and TMB, Related Fig. 3.

**a,** Neoantigen landscape of combined NSCLC and melanoma ICB cohort and comparison of neoantigen frequency. **b,** The number of candidate neoantigens originating from each SNV or an indel observed to be significantly higher than those from a fusion gene in NSCLC and melanoma external datasets. **c,** Upper panel, Kaplan-Meier (KM) curves for relapse-free survivals according to NEO2IS (left) and TMB (right) in NSCLC and melanoma ICB cohorts; bottom panel, KM curves for overall survivals stratified by NEO2IS (left) and TMB (right) in two ICB cohorts (n=156). NEO2IS > median value and TMB > upper 20% of cohort TMB used as cut-off points.

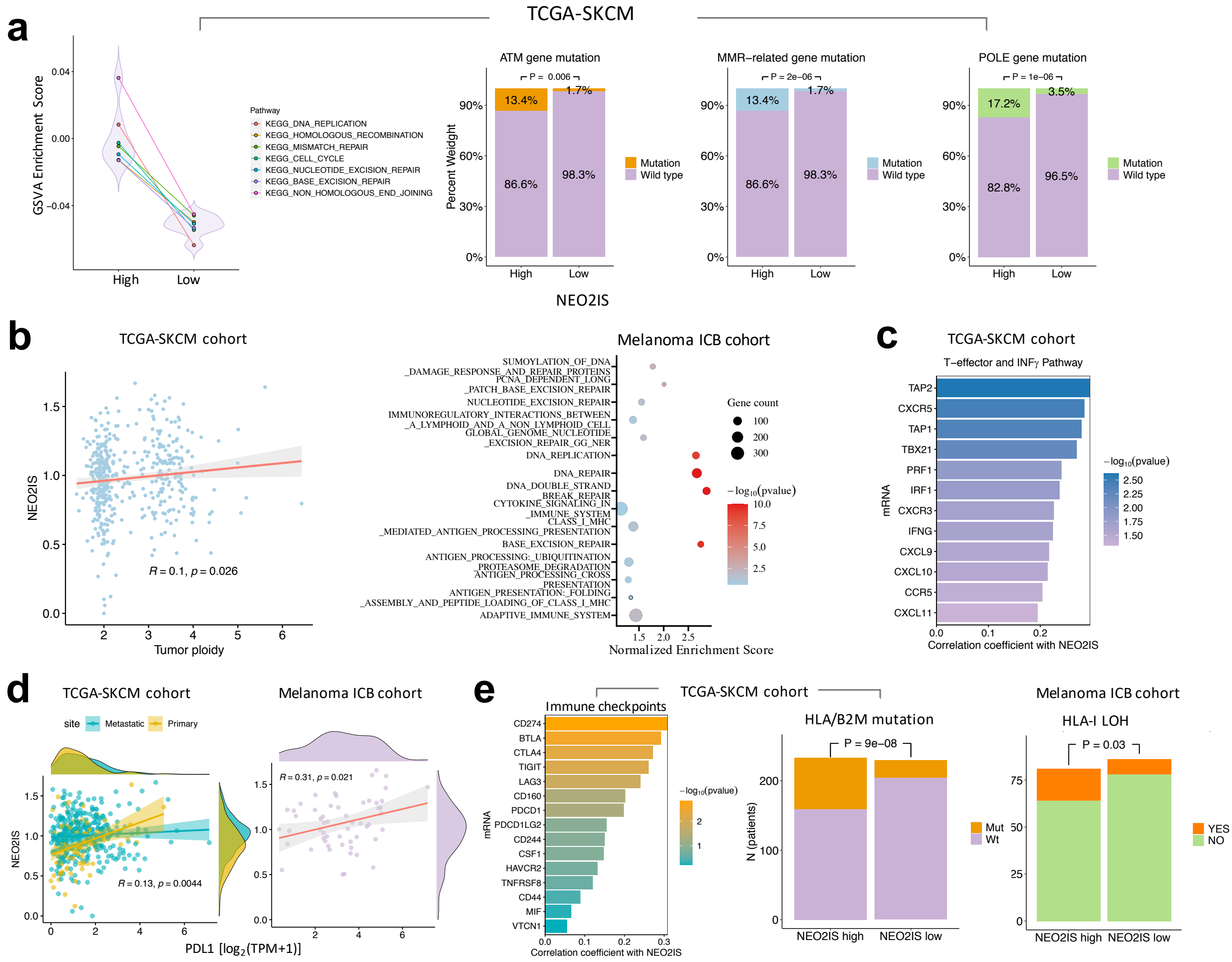


**Supplementary Fig. 4.** Genomic and molecular features associated with NEO2IS.

**a,** Left panel, GSVA showing DDR pathways significantly enriched in high NEO2IS group (adjusted p-value < 0.05); right panel, the estimated proportion of ATM, POLE and MMR-related gene mutations in two subgroups divided by median NEO2IS. **b,** Left panel, tumor ploidy significantly related to the neoantigen signature; right panel, GSEA enrichment results (q-value <0.3) for immunomodulatory REACTOME pathways (top 10) that correlated with NEO2IS in external melanoma samples. **c,** Comparison of the mRNA expressions of genes associated with T-effector and INFγ pathway between high and low NEO2IS groups. **d,** NEO2IS shows significant association with PDL1 expression levels (left panel); higher levels of co-inhibitory receptors, LOHHLA and HLA/B2M gene mutations associated with higher NEO2IS (right panel) in melanomas.

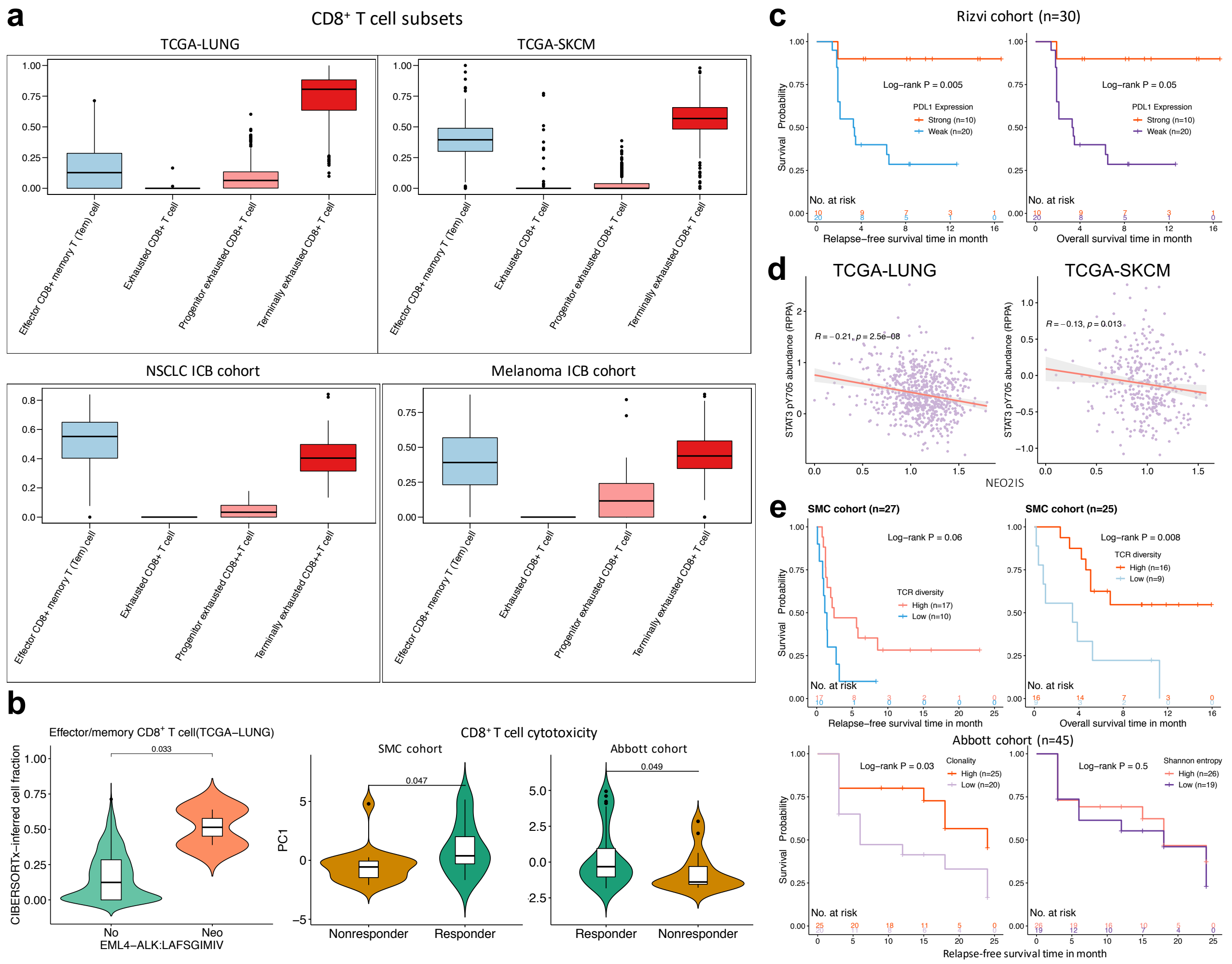


**Supplementary Fig. 5. a,** CIBERSORTx-estimated cell fractions of CD8⁺ T-cell subsets in bulk RNA-seq datasets. **b,** EML4-ALK-derived LAFSGIMIV with high neoantigen score manifests potential immunogenicity in TCGA-LUNG (left); stronger cytolytic activity observed in responders than nonresponders in NSCLC and melanoma cohorts (middle and right). **c,** Tumors with higher PDL1 expressions exhibit durable clinical response to anti-PD1 treatment (left) and longer overall survival (right) in Rizvi cohort. **d,** NEO2IS correlated significantly with STAT3 pY705 abundance in TCGA tumors. **e,** Kaplan–Meier plots for RFS and OS from SMC and Abbott cohorts stratified by TCR Shannon entropy (upper) and TCR clonality (bottom).

# Reference


1 Thorsson V, Gibbs DL, Brown SD, Wolf D, Bortone DS, Ou Yang T-H *et al*. The Immune Landscape of Cancer. *Immunity* 2018; 48: 812-830.e814.

2 Jang YE, Jang I, Kim S, Cho S, Kim D, Kim K *et al*. ChimerDB 4.0: an updated and expanded database of fusion genes. *Nucleic Acids Res* 2020; 48: D817-D824.

3 Jung H, Kim HS, Kim JY, Sun J-M, Ahn JS, Ahn M-J *et al*. DNA methylation loss promotes immune evasion of tumours with high mutation and copy number load. *Nat Commun* 2019; 10: 4278.

4 Kim JY, Choi JK, Jung H. Genome-wide methylation patterns predict clinical benefit of immunotherapy in lung cancer. *Clin Epigenetics* 2020; 12: 119.

5 Abbott CW, Boyle SM, Pyke RM, McDaniel LD, Levy E, Navarro FCP *et al*. Prediction of Immunotherapy Response in Melanoma through Combined Modeling of Neoantigen Burden and Immune-Related Resistance Mechanisms. *Clin Cancer Res* 2021; 27: 4265-4276.

6 Rizvi NA, Hellmann MD, Snyder A, Kvistborg P, Makarov V, Havel JJ *et al*. Cancer immunology. Mutational landscape determines sensitivity to PD-1 blockade in non-small cell lung cancer. *Science* 2015; 348: 124-128.

7 Bolger AM, Lohse M, Usadel B. Trimmomatic: a flexible trimmer for Illumina sequence data. *Bioinformatics* 2014; 30: 2114-2120.

8 Li H, Handsaker B, Wysoker A, Fennell T, Ruan J, Homer N *et al*. The Sequence Alignment/Map format and SAMtools. *Bioinformatics* 2009; 25: 2078-2079.

9 Li H, Durbin R. Fast and accurate long-read alignment with Burrows-Wheeler transform. *Bioinformatics* 2010; 26: 589-595.

10 Van der Auwera GA, Carneiro MO, Hartl C, Poplin R, Del Angel G, Levy-Moonshine A *et al*. From FastQ data to high confidence variant calls: the Genome Analysis Toolkit best practices pipeline. *Curr Protoc Bioinformatics* 2013; 43.

11 Cibulskis K, Lawrence MS, Carter SL, Sivachenko A, Jaffe D, Sougnez C *et al*. Sensitive detection of somatic point mutations in impure and heterogeneous cancer samples. *Nat Biotechnol* 2013; 31: 213-219.

12 Yang H, Wang K. Genomic variant annotation and prioritization with ANNOVAR and wANNOVAR. *Nat Protoc* 2015; 10: 1556-1566.

13 Lek M, Karczewski KJ, Minikel EV, Samocha KE, Banks E, Fennell T *et al*. Analysis of protein-coding genetic variation in 60,706 humans. *Nature* 2016; 536: 285-291.

14 Auton A, Brooks LD, Durbin RM, Garrison EP, Kang HM, Korbel JO *et al*. A global reference for human genetic variation. *Nature* 2015; 526: 68-74.

15 Seed G, Yuan W, Mateo J, Carreira S, Bertan C, Lambros M *et al*. Gene Copy Number Estimation from Targeted Next-Generation Sequencing of Prostate Cancer Biopsies: Analytic Validation and Clinical Qualification. *Clin Cancer Res* 2017; 23: 6070-6077.

16 Carter SL, Cibulskis K, Helman E, McKenna A, Shen H, Zack T *et al*. Absolute quantification of somatic DNA alterations in human cancer. *Nat Biotechnol* 2012; 30: 413-421.

17 McGranahan N, Rosenthal R, Hiley CT, Rowan AJ, Watkins TBK, Wilson GA *et al*. Allele-Specific HLA Loss and Immune Escape in Lung Cancer Evolution. *Cell* 2017; 171: 1259-1271.e1211.

18 Yang W, Lee K-W, Srivastava RM, Kuo F, Krishna C, Chowell D *et al*. Immunogenic neoantigens derived from gene fusions stimulate T cell responses. *Nat Med* 2019; 25: 767-775.

19 Dobin A, Davis CA, Schlesinger F, Drenkow J, Zaleski C, Jha S *et al*. STAR: ultrafast universal RNA-seq aligner. *Bioinformatics* 2013; 29: 15-21.

20 Haas BJ, Dobin A, Stransky N, Li B, Yang X, Tickle T *et al*. STAR-Fusion: Fast and Accurate Fusion Transcript Detection from RNA-Seq. *bioRxiv* 2017: 120295.

21 Uhrig S, Ellermann J, Walther T, Burkhardt P, Fröhlich M, Hutter B *et al*. Accurate and efficient detection of gene fusions from RNA sequencing data. *Genome Res* 2021; 31: 448-460.

22 Chen S, Liu M, Huang T, Liao W, Xu M, Gu J. GeneFuse: detection and visualization of target gene fusions from DNA sequencing data. *Int J Biol Sci* 2018; 14: 843-848.

23 Kim P, Tan H, Liu J, Lee H, Jung H, Kumar H *et al*. FusionGDB 2.0: fusion gene annotation updates aided by deep learning. *Nucleic Acids Res* 2022; 50: D1221-d1230.
